# A flocking algorithm for multi-agent systems with connectivity preservation under hybrid metric-topological interactions

**DOI:** 10.1371/journal.pone.0192987

**Published:** 2018-02-20

**Authors:** Chenlong He, Zuren Feng, Zhigang Ren

**Affiliations:** 1 State Key Laboratory for Manufacturing System Engineering, Systems Engineering Institute, Xi’an Jiaotong University, Xi’an, China; 2 Autocontrol Research Institute, Xi’an Jiaotong University, Xi’an, China; Washington State University, UNITED STATES

## Abstract

In this paper, we propose a connectivity-preserving flocking algorithm for multi-agent systems in which the neighbor set of each agent is determined by the hybrid metric-topological distance so that the interaction topology can be represented as the range-limited Delaunay graph, which combines the properties of the commonly used disk graph and Delaunay graph. As a result, the proposed flocking algorithm has the following advantages over the existing ones. First, range-limited Delaunay graph is sparser than the disk graph so that the information exchange among agents is reduced significantly. Second, some links irrelevant to the connectivity can be dynamically deleted during the evolution of the system. Thus, the proposed flocking algorithm is more flexible than existing algorithms, where links are not allowed to be disconnected once they are created. Finally, the multi-agent system spontaneously generates a regular quasi-lattice formation without imposing the constraint on the ratio of the sensing range of the agent to the desired distance between two adjacent agents. With the interaction topology induced by the hybrid distance, the proposed flocking algorithm can still be implemented in a distributed manner. We prove that the proposed flocking algorithm can steer the multi-agent system to a stable flocking motion, provided the initial interaction topology of multi-agent systems is connected and the hysteresis in link addition is smaller than a derived upper bound. The correctness and effectiveness of the proposed algorithm are verified by extensive numerical simulations, where the flocking algorithms based on the disk and Delaunay graph are compared.

## 1 Introduction

Flocking motion of a large group of interacting individuals is a ubiquitous phenomenon in nature [1]. All the individuals move in the same direction, keep constant relative distances, form a cohesive group, and avoid collisions with each other. Over the past two decades, researchers in control science have drawn inspiration from such biological populations and designed many flocking algorithms for multi-agent systems to benefit a variety of engineering applications such as the organization of distributed wireless networks, the cooperation of autonomous robots and the formation of unmanned vehicles [2–5].

To mimic the flocking behavior of birds, Reynolds [6] developed a flocking model, consisting of three heuristic rules known as alignment, separation, and cohesion, to generate a computer animation of bird flocks. Vicsek et al. [7] proposed a physical model to investigate the alignment of the moving directions of self-propelled particles. Regarded as a simplified Reynolds model, the Vicsek model can achieve velocity consensus if all the particles can sufficiently exchange their information as the group evolves. To obtain the sufficient condition for velocity consensus of multi-agent systems, Jadbabaie et al. [8] regarded the Vicsek model as a switched linear system and proved that the moving directions of all the agents will eventually converge to the same value if the interaction topology, which describes the relationship of information exchanges among agents in the system, is jointly connected over infinite sequences of bounded time intervals. It is well known that the realization of the multi-agent coordination critically relies on the connectivity of the interaction topology [9, 10].

Based on the three heuristic rules in the Reynolds model and some connectivity conditions, a number of flocking algorithms have been developed. Tanner et al. [11, 12] proposed two flocking algorithms for a second-order multi-agent system with either a fixed or dynamic interaction topology. The flocking controller is composed of a term of velocity consensus used to align the moving directions of agents and a gradient term of a collection of potential functions used to generate separation and cohesion behaviors. A multi-agent system steered by these algorithms can achieve flocking motion under the assumption that the interaction topology is always connected during the evolution of the system. However, this assumption is hardly satisfied in practice due to the limited sensing or communication ability of agents [13]. To discard the impractical assumption, Zavlanos et al. [14] developed a connectivity-preserving flocking algorithm, where a well-defined potential function is applied to generate a large enough force to maintain the existing edge between two adjacent agents when it is going to break. Thus, the connectivity of the interaction topology of the multi-agent system is preserved if it is connected initially. Moreover, a hysteresis in link addition is introduced as in [15] to prevent the potential function from reaching infinity so that the stability of the connectivity-preserving flocking algorithm is ensured. Along with this research direction, some improvements on this connectivity-preserving flocking algorithm have been made, such as using a bounded potential function [16], a nonlinear inter-coupling function [17], and only position measurements [18]. However, in existing flocking algorithms with the connectivity-preserving mechanism, the link between two adjacent agents, i.e., the edge in the interaction topology, cannot be disconnected once it is created. Consequently, the interaction topology of a multi-agent system may become so dense that the cost of the information exchange among agents is greatly increased as the system evolves. In addition, redundant edges may hinder the realization of a regular configuration of the multi-agent system. To generate a regular quasi-lattice formation, an additional constraint had to be imposed on the ratio of the sensing range of each agent to the desired distance between two adjacent agents [19].

To remedy the aforementioned defects of existing flocking algorithms, we propose a novel flocking algorithm, where the adjacency relationship among agents is determined by the hybrid metric-topological distance [20]. Hence, the interaction topology of the multi-agent system is represented as the range-limited Delaunay graph rather than the commonly used disk graph or Delaunay graph. Although the range-limited Delaunay graph has been applied in [21] to represent the interaction topology of self-propelled particles successfully, it is first introduced into the flocking algorithm to define the interactions among agents. Similar to the flocking algorithm proposed in [14], our algorithm consists of a term of velocity consensus and a gradient term of a collection of connectivity-preserving potential functions. Also, a hysteresis is introduced when a new link is created between two agents.

As a result, the proposed flocking algorithm has some advantages over existing ones as follows. First, due to the interaction topology represented as the range-limited Delaunay graph, the number of edges in it is usually less than that in the disk graph. Some edges can be dynamically removed from the interaction topology without destroying its connectivity as the system evolves. Consequently, the cost of the information exchange among agents in the flocking algorithm is greatly reduced and the flexibility of the flocking algorithm is increased. Second, due to the planarity of the range-limited Delaunay graph, the configuration of the multi-agent system can spontaneously converge to a more regular quasi-lattice formation without imposing the extra constraint on the sensing range of agents and desired distance between agents. Finally, the proposed flocking algorithm can be implemented in a distributed manner since the local information of agents in the limited sensing range is sufficient for each agent to construct its range-limited Delaunay neighbor set [22].

To get insights into the proposed flocking algorithm, we prove that the flocking motion of a multi-agent system can be achieved as long as its initial interaction topology is connected and the value of the hysteresis in link addition is smaller than a derived upper bound. Moreover, the theoretical superiority of the proposed flocking algorithm is verified by a variety of numerical simulations, where our flocking algorithm is compared with those based on the disk and Delaunay graph, respectively.

The remainder of this paper is organized as follows. Some preliminaries about graph theory used to describe the flocking algorithm are given in Section 2. The representation of the interaction topology, formulation of the flocking algorithm, and the stability analysis are provided in Section 3. The results of extensive numerical simulations are illustrated in Section 4. Finally, conclusions are drawn in Section 5.

## 2 Preliminaries

The interaction topology of a multi-agent system can be readily described by an undirected graph G(t)=(V,E(t)), where the vertex set V={1,2,…,N} indexes all the agents and the time-varying edge set E(t)={(i,j)∈V×V:i∼j} indicates the adjacency relationship between two agents. The cardinality of vertex and edge set denoted by |V|=N and |E(t)| are generally used to represent the size and the interaction complexity of a multi-agent system, respectively. An undirected graph is connected if, for any vertex pair, there exists a path, i.e., a sequence of distinct vertices, such that consecutive vertices between these two vertices are adjacent.

Besides the aforementioned graph-theoretic representations, the algebraic ones are also useful in the formulation of flocking algorithms, where three kinds of matrices are generally employed. The adjacency matrix *A* = [*a*_*ij*_] is a square matrix with *a*_*ij*_ = 1 if vertex *i* and *j* are adjacent, and *a*_*ij*_ = 0 otherwise. Assuming that there is no self-cycle for each vertex, diagonal elements of *A* are all zeros, i.e., *a*_*ii*_ = 0. The degree matrix *D* is a diagonal matrix, whose element is the number of neighbors of each vertex. For an undirected graph, the adjacency matrix is symmetric, so is the Laplacian matrix *L* = *D* − *A*. The Kronecker product of two matrices is commonly used in the dynamics of a multi-agent system consisting of agents with high-dimensional state variables. For matrix *X* = [*x*_*ij*_]_*m*×*n*_ and *Y* = [*y*_*ij*_]_*p*×*q*_, their Kronecker product is defined in [23] as

$$\begin{array}{c} X⊗Y={\left[\begin{array}{ccc} x_{11}Y & \ldots & x_{1n}Y\\ \vdots & \ddots & \vdots \\ x_{m1}Y & \ldots & x_{mn}Y \end{array}\right]}_{mp\times nq} \end{array}$$ (1)

## 3 Description of the connectivity-preserving flocking algorithm

The flocking algorithm we proposed is described in three parts. The representation of the interaction topology is given in Section 3.1. The flocking algorithm is formulated in detail in Section 3.2. The stability of the flocking algorithm is proved in Section 3.3.

### 3.1 Representation of interaction topology of multi-agent systems

The interaction topology of a multi-agent system specifies the adjacency relationship of all the agents and can be conveniently represented by a proximity graph, in which the determination of edges between two agents is closely related to their relative positions. Different definitions of the adjacency relationship induce different kinds of proximity graphs [22].

As an agent generally has a limited sensing ability, its sensing range can be intuitively illustrated as a circle. As a result, the interaction topology of the multi-agent system consisting of agents with a limited sensing range determined by metric distance is commonly represented as the unit disk graph (Shortened to UDG). Here, in order to emphasize the limited sensing range *r*, this proximity graph is called the *r*-disk graph and denoted as $G_{disk}\left(r\right)$, where agent *i* regards agent *j* as its neighbor if agent *j* locates inside the sensing range of agent *i* and vice versa. Thus, the neighbor set of agent *i* is denoted as

$$\begin{array}{c} N_{i}\left(t\right)=\left\{\;j\in V:\right\|q_{j}-q_{i}\|<r\;\}, \end{array}$$ (2)

where *q_i_*, $q_{j}\in R^{n}$ are the positions of two agents in an *n*-dimensional space and the notation ‖ ⋅ ‖ represents the Euclidean norm of a vector. However, in the connectivity-preserving flocking algorithm, edges in the *r*-disk graph cannot be disconnected once they are created so that the number of edges in the *r*-disk graph will increase monotonously, which leads to a higher cost of information exchange among agents.

Moreover, recent research findings have shown that birds within a flock actually interact with 6 or 7 closest neighbors irrespective of their metric distances [24, 25]. This kind of the adjacency relationship is determined by the so-called topological distance. In a group, an individual will dominate a region, where the distance from any point to itself is smaller than to any other individual and interact with others whose dominant regions intersect with itself. According to this physical explanation, the interaction topology of the multi-agent system is determined by topological distance, which induces the Delaunay graph denoted as $G_{D}$. In other research fields, such as the mathematics and computational geometry, the triangulation technique used to generate the such a graph is also called the Delaunay triangulation. Its definition is closely related to its dual graph known as the Voronoi diagram, also called the Voronoi tessellation or Voronoi partition, in which multiple disjoint regions dominated by distinct agents are called the Voronoi cells of generators. The Voronoi cell associated with agent *i* is a region consisting of all the points closer to *q*_*i*_ than to others and is formulated as

$$\begin{array}{c} V_{i}=\{\;q\in R^{n}|\|q_{i}-q\|\leq \|q_{j}-q\left\|,j\neq i\;\right\}. \end{array}$$ (3)

Two agents are Delaunay neighbors if their Voronoi cells intersect. Therefore, the Delaunay neighbor set of agent *i* is denoted as

$$\begin{array}{c} N_{i}^{D}=\left\{\;j\in V:V_{i}\cap V_{j}\neq \emptyset \;\right\}. \end{array}$$ (4)

Although the Delaunay graph has been used to represent the interaction topology in biological and physical groups [26, 27] and the concept of the topological distance has been applied to design the flocking algorithm [28], it is impractical to employ the Delaunay graph to represent the interaction topology of the multi-agent system consisting of agents having a limited sensing ability since some edges beyond the sensing range of agents may appear.

In order to represent the interaction topology of the multi-agent system in a more realistic way, the metric and topological distance applied in the *r*-disk and Delaunay graph are integrated together to form a new kind of proximity graph known as the *r*-limited Delaunay graph denoted as $G_{LD}\left(r\right)$, where an agent first finds other agents in its limited sensing range and then filters out those agents whose Voronoi cells have the common boundaries with its Voronoi cell as its neighbors. In the *r*-limited Delaunay graph, two agents are adjacent if their dominant regions called the *r*/2-limited Voronoi cell intersect. Similar to the definition of the Voronoi cell, the *r*/2-limited Voronoi cell associated with agent *i* is defined as

$$\begin{array}{c} V_{i}^{r/2}=V_{i}\cap \bar{B}\left(q_{i},r/2\right), \end{array}$$ (5)

where $\bar{B}\left(q_{i},r/2\right)$ is a closed circle with the center *q*_*i*_ and radius *r*/2. The *r*-limited Delaunay neighbor set of agent *i* is denoted as

$$\begin{array}{c} N_{i}^{LD}\left(r\right)=\left\{\;j\in V:V_{i}^{r/2}\cap V_{j}^{r/2}\neq \emptyset \;\right\}. \end{array}$$ (6)

In the *r*-limited Delaunay graph, many edges appearing in the *r*-disk graph can be removed without affecting its connectivity and those conflicting with the limited sensing range of agents in the Delaunay graph do not arise. Therefore, the connectivity-preserving flocking algorithm based on this new proximity graph has a low cost of information exchanges and remains a distributed algorithm.

**Fig 1:**
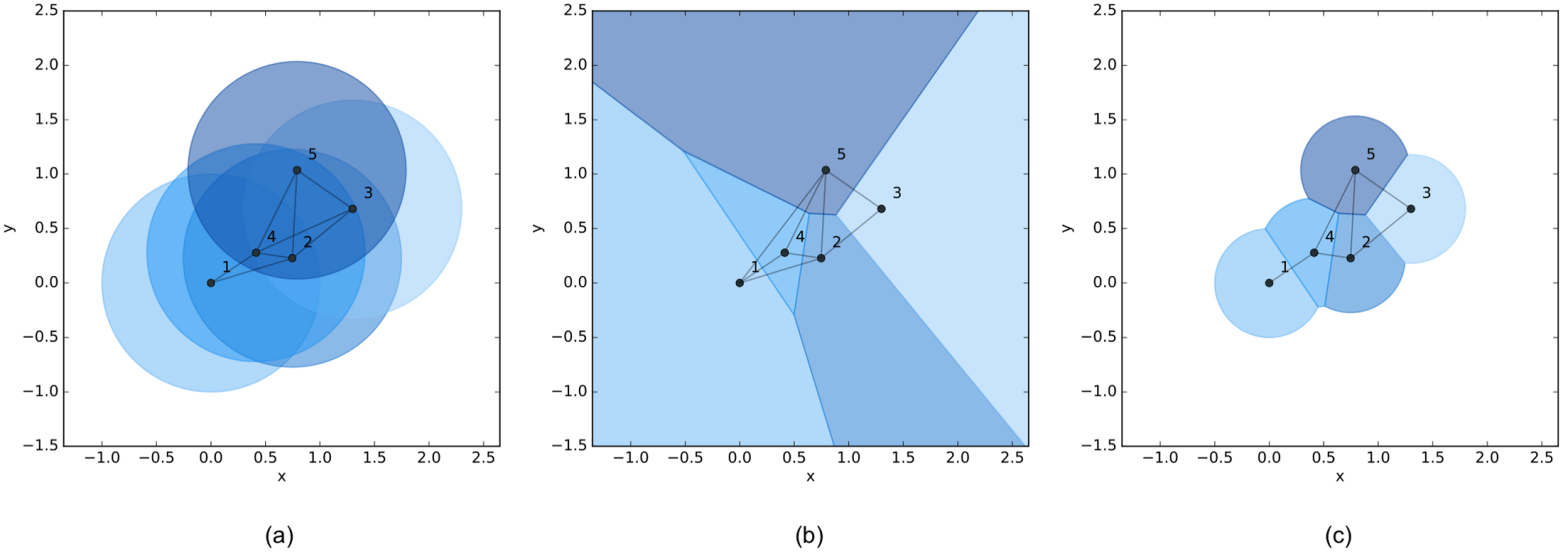
Different types of proximity graphs. The interaction topology of a multi-agent system with 5 agents represented as three types of proximity graphs. (a) *r*-disk graph. (b) Delaunay graph. (c) *r*-limited Delaunay graph.

In Fig 1, a multi-agent system with 5 agents is given and its interaction topology is represented by the three types of aforementioned proximity graphs, where the solid dots with indices represent agents in a plane, the regions filled with different colors are dominated by distinct agents and the grey lines between two agents are edges in the proximity graph. Except for the connectivity, the three proximity graphs have significant differences in the following aspects. In the *r*-disk graph, the number of edges is more than that in its two counterparts and there are some intersected edges. In the Delaunay graph, intersecting edges do not arise since the graph is planar, but some long-range edges beyond the limited sensing range of agents may appear. In contrast to above two graphs, both intersected and long-range edges are eliminated in the *r*-limited Delaunay graph so that the number of edges is much smaller than its two counterparts. Moreover, the *r*-limited Delaunay graph is spatially distributed over the *r*-disk graph [29], which means that each agent can independently compute its own *r*-limited Delaunay neighbor set based on the local information of agents in its sensing range. Consequently, the flocking algorithm based on the *r*-limited Delaunay graph as the interaction topology of a multi-agent system can be implemented in a distributed manner (see the counterexample in [30], where the interaction topology of a self-propelled particle group is represented as the Delaunay graph and the algorithm cannot be implemented in a distributed fashion).

### 3.2 Formulation of flocking algorithm

We consider a multi-agent system consisting of *N* agents. The dynamics of agent *i* can be described by a double integrator as

$$\begin{array}{c} \left\{ \begin{array}{c} {\dot{q}}_{i}=p_{i}\\ {\dot{p}}_{i}=u_{i} \end{array},\right. \end{array}$$ (7)

where *q*_*i*_, *p*_*i*_, $u_{i}\in R^{n}$ are the position, velocity, and acceleration, respectively. As the control input, the acceleration embodies the three heuristic rules in the Reynolds model and is composed of two terms as

$$\begin{array}{c} u_{i}=\alpha_{i}+\beta_{i}. \end{array}$$ (8)

**Fig 2:**
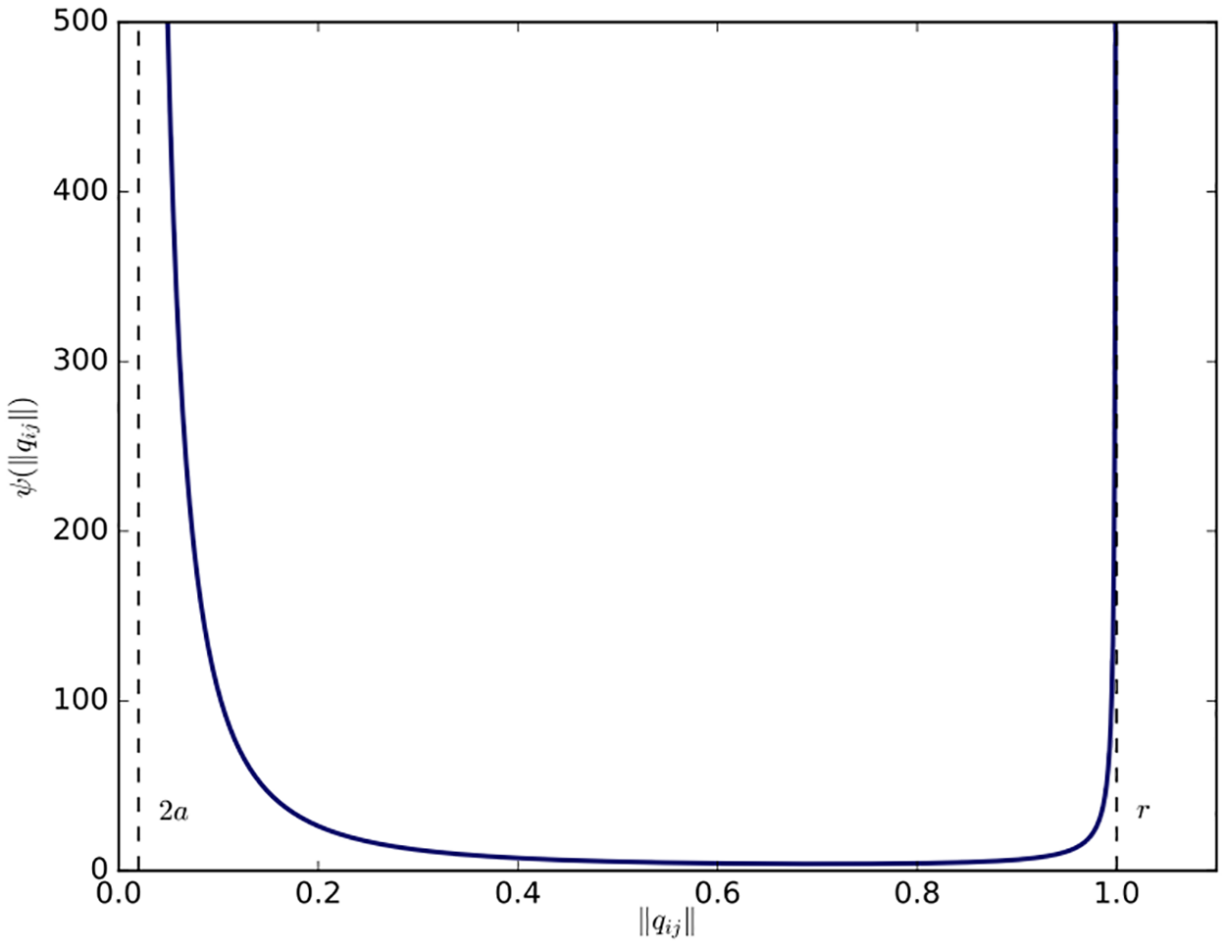
Potential function. The connectivity-preserving potential function with *r* = 1 and *a* = 0.01.

The first term

$$\begin{array}{c} \alpha_{i}=-\sum_{j\in N_{i}^{LD}\left(r\right)\left(t\right)}\left(p_{i}-p_{j}\right), \end{array}$$ (9)

corresponding to the alignment rule, is used to achieve velocity consensus since the velocity difference between agent *i* and all its *r*-limited Delaunay neighbors will gradually disappear as the system evolves. The second term

$$\begin{array}{c} \beta_{i}=-\sum_{j\in N_{i}^{LD}\left(r\right)\left(t\right)}\nabla_{q_{i}}\psi (\|q_{ij}\left\|\right), \end{array}$$ (10)

corresponding to the cohesion and separation rules, is a gradient-based force produced by a collection of well-defined potential functions *ψ*(‖*q*_*ij*_‖), where *q*_*ij*_ = *q*_*i*_ − *q*_*j*_. The potential function should be nonnegative and has a global minimum at the desired distance between two agents so that the attractive or repulsive force is generated when the relative distance between two adjacent agents is greater or less than the desired distance, respectively. Here, the potential function is defined as

$$\begin{array}{c} \psi (\|q_{ij}\left\|\right)=\frac{1}{\|q_{ij}{\|}^{2}-4a^{2}}+\frac{1}{r^{2}-{\left\|q_{ij}\right\|}^{2}}, \end{array}$$ (11)

where *r* is the sensing range of each agent as before and *a* is the radius of a small collision shell of each agent. It is obvious that the potential function has its global minimum at ‖*q*_*ij*_‖ = *d* ∈ (2*a*, *r*). As a result, the function can produce a repulsive force in the interval (2*a*, *d*) and an attractive force in (*d*, *r*). Especially, when ‖*q*_*ij*_‖ approaches 2*a* or *r*, *ψ*(‖*q*_*ij*_‖) tends to infinity to avoid collisions or maintain the existing edge between two adjacent agents. It is similar to the connectivity-preserving potential function applied in [14] other than the introduction of the collision shell (see Fig 2). The collision shell is used to ensure the connectivity of the interaction topology at the switching time and the stability of the flocking algorithm when links between non-adjacent agents are created. The rigorous proof of the connectivity and stability of the flocking algorithm will be elaborated in the next subsection.

Since the potential function with connectivity-preserving mechanism will become infinite when the distance between two agents tends to the limited sensing range *r*, in order to prevent the potential function from reaching infinity when two non-neighboring agents gradually approach each other and just become adjacent, the new link should not be created immediately but delayed for a small displacement. This method is widely applied in the flocking algorithms with a connectivity-preserving potential function [14, 15, 31].

For a multi-agent system, its interaction topology represented as the *r*-limited Delaunay graph has a time-dependent edge set with dynamical link addition and deletion as the system evolves. Due to the hysteresis, links do not be created immediately when two agents just become the *r*-limited Delaunay neighbor to ensure the boundedness of forces generated by the unbounded potential function. Thus, the *r*-limited Delaunay graph with the hysteresis in link addition $G_{LD}^{\prime }\left(r\right)\left(t\right)=\left(V,E^{\prime }\left(t\right)\right)$ is slightly different from the standard one $G_{LD}\left(r\right)\left(t\right)=\left(V,E\left(t\right)\right)$ in its time-varying edge set $E^{\prime }\left(t\right)=\left\{\left(i,j\right)|i,j\in V\right\}$ defined as

- if (i,j)∈E(0) and 2*a* < ‖*q*_*i*_(0) − *q*_*j*_(0)‖ < *r* − *ε*, then $\left(i,j\right)\in E^{\prime }\left(0\right)$;
- if $\left(i,j\right)\in E^{\prime }\left(t^{-}\right)$ and (i,j)∈E(t), then $\left(i,j\right)\in E^{\prime }\left(t\right)$;
- if $\left(i,j\right)\in E^{\prime }\left(t^{-}\right)$ and (i,j)∉E(t), then $\left(i,j\right)\notin E^{\prime }\left(t\right)$;
- if $\left(i,j\right)\notin E\left(t^{-}\right)$, (i,j)∈E(t) and ‖*q*_*i*_(*t*) − *q*_*j*_(*t*)‖ < *r* − *ε*, then $\left(i,j\right)\in E^{\prime }\left(t\right)$;
- if $\left(i,j\right)\notin E^{\prime }\left(t^{-}\right)$ and (i,j)∉E(t), then $\left(i,j\right)\notin E^{\prime }\left(t\right)$.

At the initial time *t* = 0, the length of all the edges in the *r*-limited Delaunay graph with the hysteresis in link addition is restricted in the interval (2*a*, *r* − *ε*). The hysteresis in link addition *ε* should satisfy *ε* < ε* ∈ (0, *r* − 2*a*), where ε* is the upper bound of the hysteresis in link addition and will be given in Lemma 2. During the evolution of the multi-agent system, the edge between two agents is determined by both the adjacency relationship before the switching time *t*^−^ and the relative distance between two agents at the current time *t*. An existing edge is retained if two agents are still the *r*-limited Delaunay neighbor at the current moment. A new edge is created if two non-adjacent agents become the *r*-limited Delaunay neighbor, while their distance is smaller than *r* − *ε*. Despite different from the standard edge set of the *r*-limited Delaunay graph, we still use the notation $N_{i}^{LD}\left(r\right)$ as the *r*-limited Delaunay neighbors set of agent *i* with the hysteresis in link addition throughout the paper.

### 3.3 Stability analysis of flocking algorithm

For a multi-agent system consisting of *N* agents with flocking inputs given in Eqs (8)–(10), we first represent it as a nonlinear system in the coordinates of its center of mass (shortened to COM), then judge whether it can achieve flocking motion by analyzing the stability of the equilibrium. Concretely, we denote the position and velocity of the COM of the multi-agent system as

$$\begin{array}{c} \left\{ \begin{array}{cc} \bar{q} & =\frac{1}{N}\sum_{i=1}^{N}q_{i}\\ \bar{p} & =\frac{1}{N}\sum_{i=1}^{N}p_{i} \end{array},\right. \end{array}$$ (12)

then, the relative position and velocity between agent *i* and the COM are ${\tilde{q}}_{i}=q_{i}-\bar{q}$ and ${\tilde{p}}_{i}=p_{i}-\bar{p}$, respectively. Thus, the dynamics of agent *i* in the coordinates of the COM becomes

$$\begin{array}{c} \left\{ \begin{array}{cc} {\dot{\tilde{q}}}_{i} & ={\tilde{p}}_{i}\\ {\dot{\tilde{p}}}_{i} & =-\sum_{j\in N_{i}^{LD}\left(r\right)\left(t\right)}\left({\tilde{p}}_{i}-{\tilde{p}}_{j}\right)-\sum_{j\in N_{i}^{LD}\left(r\right)\left(t\right)}\nabla_{q_{i}}\psi (\|{\tilde{q}}_{ij}\left\|\right). \end{array}\right. \end{array}$$ (13)

The total energy of the multi-agent system composed of the potential and kinetic energy of all the agents can be denoted as

$$\begin{array}{c} W\left(t\right)=\frac{1}{2}\sum_{i=1}^{N}(\sum_{j\in N_{i}^{LD}\left(r\right)\left(t\right)}\psi (\|{\tilde{q}}_{ij}\left\|\right)+{\tilde{p}}_{i}^{T}\tilde{p_{i}}). \end{array}$$ (14)

Obviously, the potential function is invariant under the change of coordinations as $\psi (\|{\tilde{q}}_{ij}\left\|\right)=\psi (\|q_{ij}\left\|\right)$ and *W*(*t*) is positive semi-definite.

Due to the movement of agents, the multi-agent system has a dynamically switched interaction topology and can be described as a switched nonlinear system taking the following form

$$\begin{array}{c} \dot{x}=f_{\sigma (t)}\left(x\right), \end{array}$$ (15)

where the state variable $x={\left[\tilde{q},\tilde{p}\right]}^{T}$ is a stacked vector with

$$\begin{array}{c} \begin{array}{cc} \tilde{q} & ={\left({\tilde{q}}_{11}^{T},\ldots ,{\tilde{q}}_{1N}^{T},\ldots ,{\tilde{q}}_{N1}^{T},\ldots ,{\tilde{q}}_{NN}^{T}\right)}^{T}\in R^{N^{2}n},\\ \tilde{p} & ={\left({\tilde{p}}_{1}^{T},\ldots ,{\tilde{p}}_{N}^{T}\right)}^{T}\in R^{Nn}, \end{array} \end{array}$$

and *f*_*σ*(*t*)_(⋅) is a nonlinear switching function. The switching signal σ:[0,∞)→P is piecewise constant and continuous from the right. The finite set P contains indices of all the possible *r*-limited Delaunay graphs. Assume that switchings happen at time *t*_*k*_ (*k* = 0, 1, …) and the interaction topology is fixed between any two consecutive switching times, i.e., in the interval [*t*_*k*−1_, *t*_*k*_). At the switching time, link addition and deletion occur in the edge set of the *r*-limited Delaunay graph. Before proving the stability of the flocking algorithm, some necessary results about the connectivity of the interaction topology at the switching time will be given first in the following two lemmas.

**Lemma 1.** *For a multi-agent system employing the r-limited Delaunay graph to represent its interaction topology and the connectivity-preserving potential function in its flocking inputs, the connectivity of the interaction topology can be preserved at the switching time if it is connected before switching and the value of hysteresis in link addition is smaller than a derived upper bound*.

**Fig 3:**
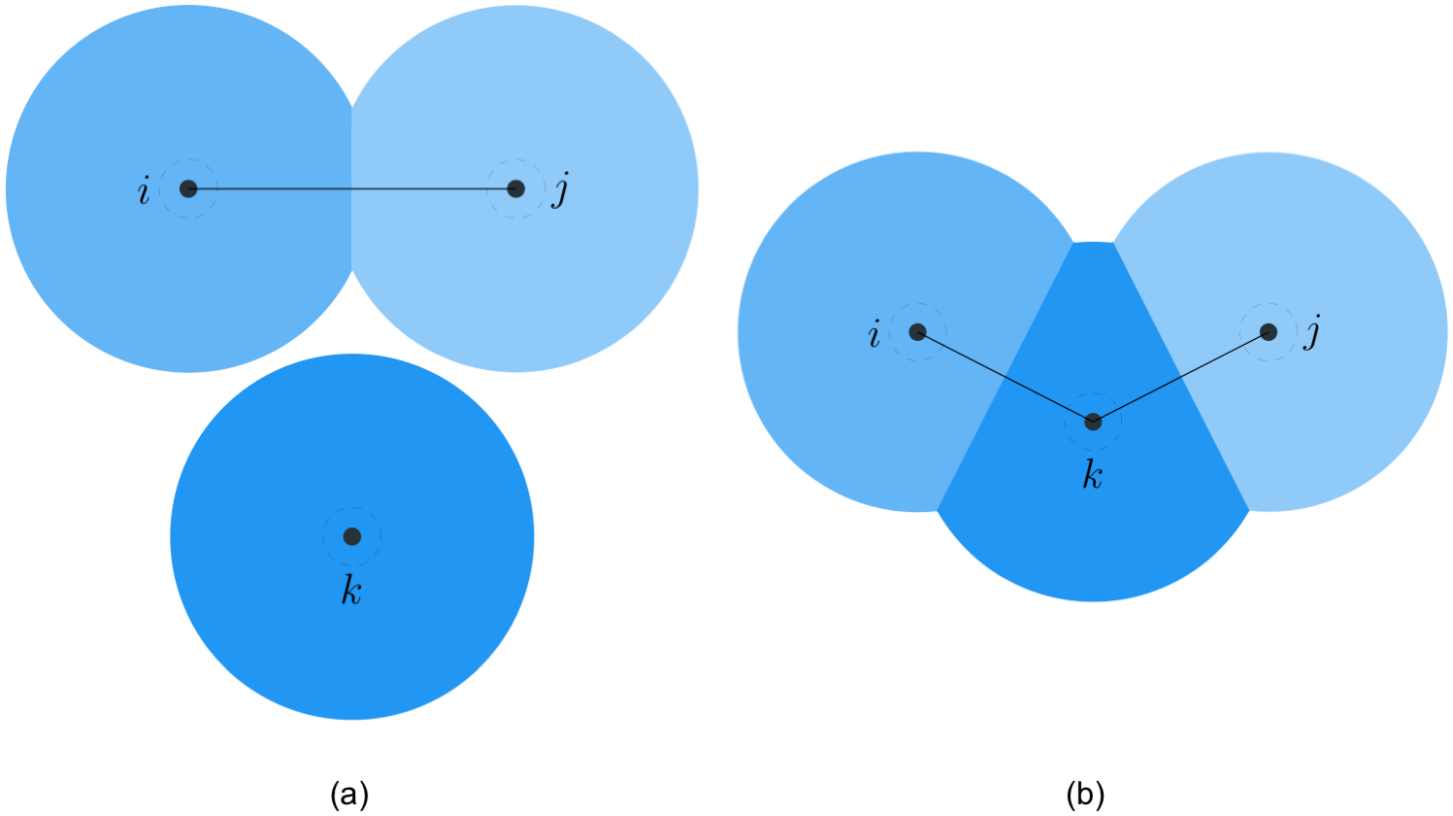
The first switching case. The case of replacing one existing link by two new links at the switching time. (a) before switching. (b) after switching.

**Fig 4:**
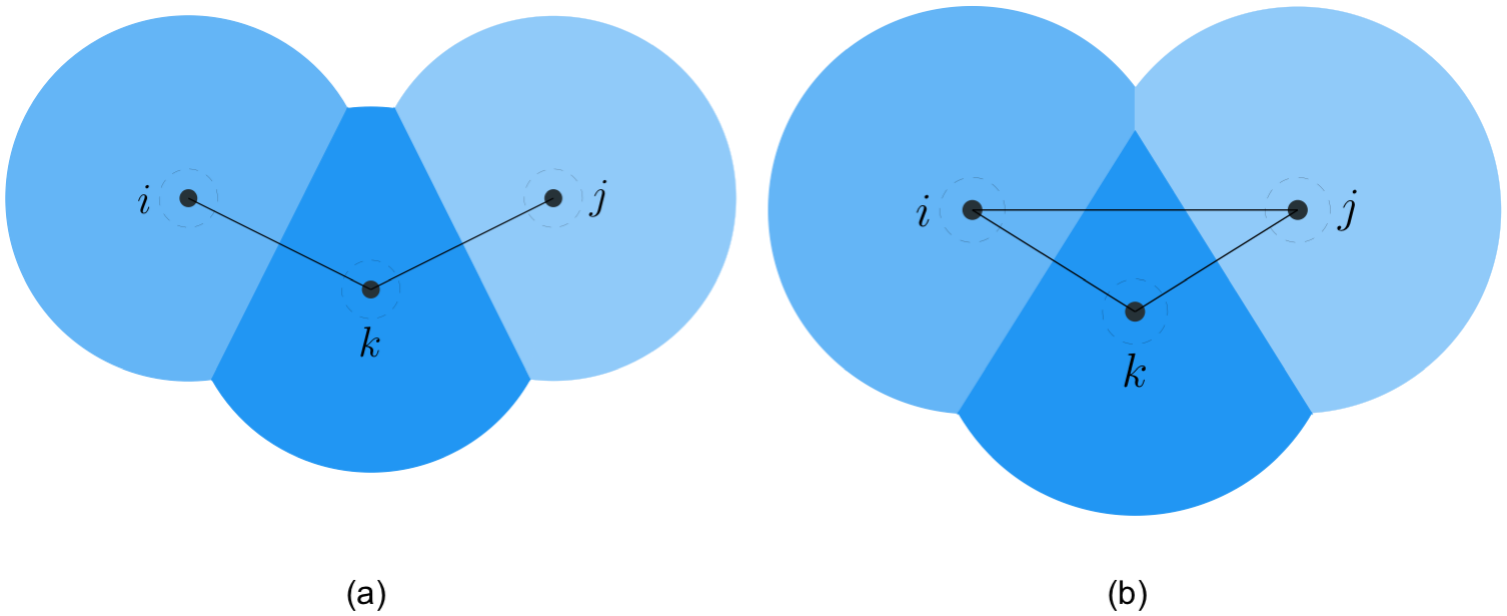
The second switching case. The case of creating one link at the switching time. (a) before switching. (b) after switching.

**Fig 5:**
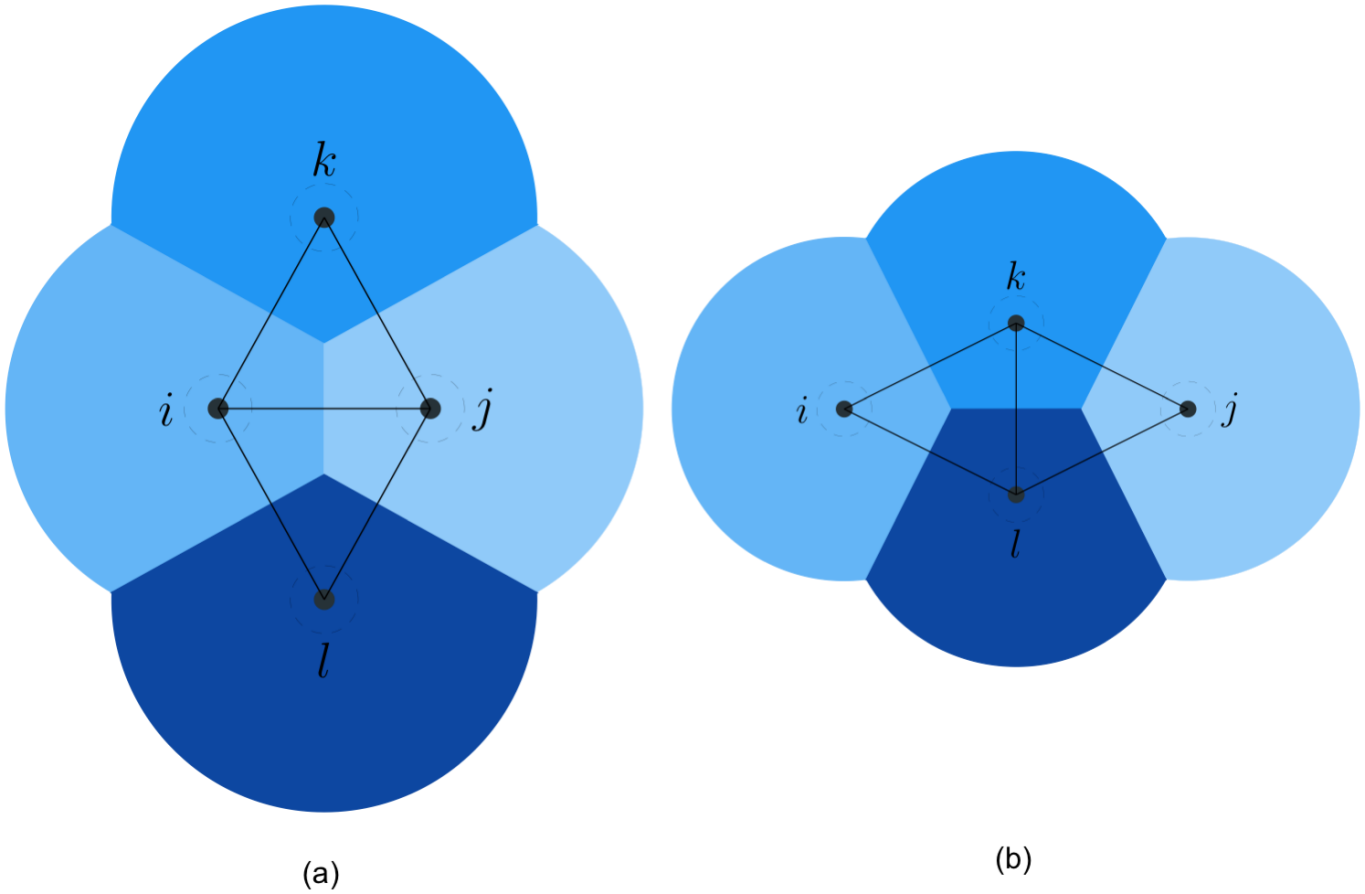
The third switching case. The case of flipping diagonals at the switching time. (a) before switching. (b) after switching.

*Proof*. Since the interaction topology represented as the *r*-limited Delaunay graph is a connected graph before switching, three cases may happen at the switching time. The first case is shown in Fig 3, where a single agent *k* indirectly connected to another two adjacent agents *i* and *j* moves towards them, then the existing link (*i*, *j*) will be replaced by two new links (*i*, *k*) and (*j*, *k*). Due to the hysteresis in link addition, the connectivity of the interaction topology can be preserved if two new links are created in time before the existing link is deleted. This case will be specially analyzed in the following lemma, where the upper bound of the hysteresis is derived in detail. In the second case illustrated in Fig 4, only one new link (*i*, *j*) is created between two non-adjacent agents. Undoubtedly, link addition does not affect the connectivity of the interaction topology. The last case is known as flipping diagonals in the construction algorithm of the Delaunay graphs [32]. In Fig 5, four agents form a quadrilateral shape and are connected by four sides (*i*, *k*), (*i*, *l*), (*j*, *k*), (*j*, *l*) and one of the two diagonals (*k*, *l*), for instance. Since two diagonals are redundant for the connectivity, replacing (*k*, *l*) with (*i*, *j*) does not affect the connectivity of the interaction topology. In a word, the connectivity of the interaction topology can be preserved at each switching time.

*Remark* 1. In the case of flipping diagonals, one of the two diagonals is deleted and its counterpart may not be created immediately due to the hysteresis in link addition. Then, the number of edges in the interaction topology will decrease temporarily. However, this situation will disappear after a transient process as the system evolves and does not destroy the connectivity of the interaction topology and the stability of the multi-agent system.

Although the value of the hysteresis in link addition can be arbitrarily chosen as a small value in existing flocking algorithms, a too large hysteresis may destroy the connectivity of the interaction topology represented as the *r*-limited Delaunay graph in the proposed flocking algorithm. As mentioned in the first case of the proof of Lemma 1, if existing links have been deleted but new links have not yet been created in time due to the hysteresis in link addition, the inconsistency of the connectivity will occur. In order to avoid this situation, a derived upper bound of the hysteresis in link addition is used to guarantee that new links can be created in time so that the interaction topology still remains connected.

By introducing a collision shell into each agent, the worst case in link addition is used to determine the upper bound of the hysteresis. The derivation of this value is given in the following lemma.

**Lemma 2.** *At the switching time, the connectivity of the interaction topology will not be destroyed by the hysteresis in link addition if its value ε is set to be smaller than an upper bound* $\varepsilon^{*}=r-\sqrt{r^{2}-4a^{2}}$.

**Fig 6:**
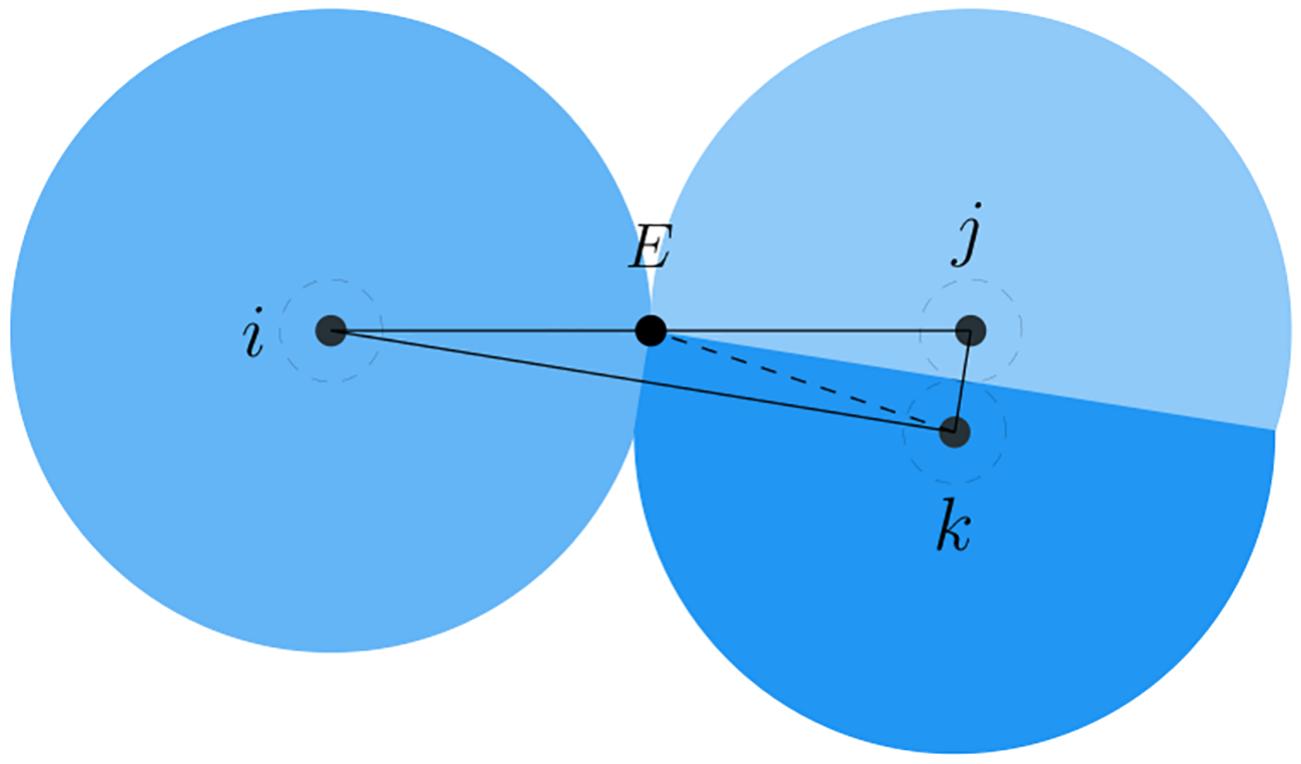
The worst case of link deletion. The critical configuration used to determine the upper bound of the hysteresis in link addition.

*Proof*. The determination of the upper bound of the hysteresis in link addition is a geometric problem. Without loss of generality, the configuration of 3 agents in the worst case is illustrated in Fig 6, where only agent *i* and *j* are adjacent before switching. Then, agent *k* approaches the adjacent pair and lies in the middle of them. The existing link (*i*, *j*) is replaced by two new links (*i*, *k*) and (*j*, *k*). Therefore, the length of the new links, in this case, is used to determine the upper bound of the hysteresis in link addition.

According to the relative distances among agents shown in Fig 6, the distance between agent *i* and *j* is slightly smaller than the sensing range *r* and that between *j* and *k* is about 2*a* because of the collision shell. In this situation, the distance between *i* and *k* is the critical distance associated with the connectivity of the interaction topology. Consequently, the upper bound of the hysteresis in link addition is derived as follows. First, connect the middle point between *i* and *j* denoted as *E* to *k* by a dashed helping line. It is evident that *E* is the center of the circumcircle through three agents since distances from *E* to the three agents are all equal to *r*/2. Then, the distance between *i* and *k* can be easily calculated as

$$\begin{array}{c} d_{ik}=\sqrt{d_{ij}^{2}-4a^{2}}=\sqrt{r^{2}-4a^{2}}. \end{array}$$ (16)

At the current distance, the new link (*i*, *k*) must be created, otherwise, the interaction topology is not connected anymore. Finally, the upper bound of the hysteresis in link addition is given by

$$\begin{array}{c} d_{ik}=r-\varepsilon^{*}\Rightarrow \varepsilon^{*}=r-\sqrt{r^{2}-4a^{2}}. \end{array}$$ (17)

Once the value of the hysteresis in link addition is restricted by this upper bound at the switching time, the connectivity of the interaction topology can be preserved anyway. Notably, the introduction of the collision shell ensures that the upper bound of the hysteresis in link addition is a finite value, otherwise, it will be zero.

*Remark* 2. Due to the hysteresis in link addition, the interaction topology may be represented as a subgraph of the exact *r*-limited Delaunay graph, but the connectivity and the planarity remain unchanged.

Based on above two lemmas, the main result of this section is stated in the following theorem.

**Theorem 1.** *For a multi-agent system consisting of N agents, each of which is modeled by a double integrator in* Eq (7) *and steered by the flocking inputs in* Eq (8), *assume that the initial interaction topology* *G*_*LD*_(*r*)(*t*_0_) *is connected, the initial energy* $W\left(t_{0}\right)=W\left(\tilde{q}\left(t_{0}\right),\tilde{p}\left(t_{0}\right)\right)$ is finite, and the value of the hysteresis in link addition satisfies ε < ε*. Then, the following statements hold:

1. *G*_*LD*_(*r*)(*t*) *is connected for all t* > 0;
2. *All the agents asymptotically move with the same velocity*;
3. *Almost every final configuration locally minimizes each agent’s collective potential function* $\sum_{j\in N_{i}^{LD}\left(r\right)}\psi (\|q_{ij}\left\|\right)$;
4. *No inter-agent collisions occur for all t* > 0.

*Proof*. Since the connectivity of the interaction topology at the switching time *t*_*k*_ (*k* = 0, 1, …) has been proved in Lemma 1, we just need to analyze the connectivity of the interaction topology between two consecutive switching times, i.e., in each time interval [*t*_*k*−1_, *t*_*k*_), to complete the proof of statement 1. Without loss of generality, we assume that the initial time *t*_0_ = 0. Under the condition that the initial interaction topology *G*_*LD*_(*r*)(*t*_0_) is connected and the initial energy *W*(*t*_0_) is finite, the time derivative of the total energy *W*(*t*) in [*t*_0_, *t*_1_) is

$$\begin{array}{c} \begin{array}{cc} \dot{W}\left(t\right) & =\frac{1}{2}\sum_{i=1}^{N}\sum_{j\in N_{i}^{LD}\left(r\right)}\dot{\psi }\left(\right\|{\tilde{q}}_{ij}\left\|\right)\\ & -\sum_{i=1}^{N}{\tilde{p}}_{i}^{T}(\sum_{j\in N_{i}^{LD}\left(r\right)}\left({\tilde{p}}_{i}-{\tilde{p}}_{j}\right)-\sum_{j\in N_{i}^{LD}\left(r\right)}\nabla_{{\tilde{q}}_{i}}\psi (\|{\tilde{q}}_{ij}\left\|\right)). \end{array} \end{array}$$ (18)

Due to the fact that

$$\begin{array}{c} \frac{\partial \psi (\|{\tilde{q}}_{ij}\|)}{\partial {\tilde{q}}_{ij}}=\frac{\partial \psi (\|{\tilde{q}}_{ij}\|)}{\partial {\tilde{q}}_{i}}=-\frac{\partial \psi (\|{\tilde{q}}_{ij}\|)}{\partial {\tilde{q}}_{j}}, \end{array}$$

the first term on the right of Eq (18) is simplified as

$$\begin{array}{c} \begin{array}{cc} \frac{1}{2}\sum_{i=1}^{N}\sum_{j\in N_{i}^{LD}\left(r\right)\left(t\right)}\dot{\psi }\left(\right\|{\tilde{q}}_{ij}\left\|\right) & =\frac{1}{2}\sum_{i=1}^{N}\sum_{j\in N_{i}^{LD}\left(r\right)\left(t\right)}{\dot{\tilde{q}}}_{ij}^{T}\nabla_{{\tilde{q}}_{ij}}\psi (\|{\tilde{q}}_{ij}\left\|\right)\\ & =\frac{1}{2}\sum_{i=1}^{N}\sum_{j\in N_{i}^{LD}\left(r\right)\left(t\right)}({\dot{\tilde{q}}}_{i}^{T}\nabla_{{\tilde{q}}_{ij}}\psi (\|{\tilde{q}}_{ij}\left\|\right)-{\dot{\tilde{q}}}_{j}^{T}\nabla_{{\tilde{q}}_{ij}}\psi (\|{\tilde{q}}_{ij}\left\|\right))\\ & =\frac{1}{2}\sum_{i=1}^{N}\sum_{j\in N_{i}^{LD}\left(r\right)\left(t\right)}({\dot{\tilde{q}}}_{i}^{T}\nabla_{{\tilde{q}}_{i}}\psi (\|{\tilde{q}}_{ij}\left\|\right)+{\dot{\tilde{q}}}_{j}^{T}\nabla_{{\tilde{q}}_{j}}\psi (\|{\tilde{q}}_{ij}\left\|\right))\\ & =\sum_{i=1}^{N}\sum_{j\in N_{i}^{LD}\left(r\right)\left(t\right)}{\dot{\tilde{q}}}_{i}^{T}\nabla_{{\tilde{q}}_{i}}\psi (\|{\tilde{q}}_{ij}\left\|\right). \end{array} \end{array}$$ (19)

Then, the derivative of *W*(*t*) becomes

$$\begin{array}{c} \begin{array}{cc} \dot{W}\left(t\right) & =\sum_{i=1}^{N}\sum_{j\in N_{i}^{LD}\left(r\right)\left(t\right)}{\tilde{p}}_{i}^{T}\nabla_{{\tilde{q}}_{i}}\psi (\|{\tilde{q}}_{ij}\left\|\right)-\sum_{i=1}^{N}{\tilde{p}}_{i}^{T}\sum_{j\in N_{i}^{LD}\left(r\right)\left(t\right)}\left({\tilde{p}}_{i}-{\tilde{p}}_{j}\right)\\ & -\sum_{i=1}^{N}\sum_{j\in N_{i}^{LD}\left(r\right)\left(t\right)}{\tilde{p}}_{i}^{T}\nabla_{{\tilde{q}}_{i}}\psi (\|{\tilde{q}}_{ij}\left\|\right)\\ & =-{\tilde{p}}^{T}\left(L_{G_{LD}\left(r\right)}⊗I_{n}\right)\tilde{p}\leq 0, \end{array} \end{array}$$ (20)

where $L_{G_{LD}\left(r\right)}$ is the Laplacian matrix of $G_{LD}\left(r\right)$. Due to the positive semi-definiteness of $L_{G_{LD}\left(r\right)}$, the derivative of *W*(*t*) is nonpositive, which implies that

$$\begin{array}{c} W\left(t\right)\leq W\left(0\right)<\infty ,\;\text{for}\;t\in \left[t_{0},t_{1}\right). \end{array}$$ (21)

Similarly, the time derivative of *W*(*t*) in the subsequent interval [*t*_*k*−1_, *t*_*k*_) is

$$\begin{array}{c} \dot{W}\left(t\right)=-{\tilde{p}}^{T}\left(L_{G_{LD}\left(r\right)}⊗I_{n}\right)\tilde{p}\leq 0, \end{array}$$ (22)

which implies that

$$\begin{array}{c} W\left(t\right)\leq W\left(t_{k-1}\right)<\infty ,\;\text{for}\;t\in \left[t_{k-1},t_{k}\right). \end{array}$$ (23)

Therefore, no existing edge is lost in all the time intervals so that $G_{LD}\left(r\right)\left(t\right)$ is always connected for all *t* > 0. This completes the proof of statement 1.

Under the condition of the connected interaction topology, we next prove statement 2. First of all, we demonstrate the number of switchings in the interaction topology is finite. For the first two cases mentioned in Lemma 1, the number of link addition is greater than that of link deletion so that the number of edges in the interaction topology increases. Due to the planarity of the *r*-limited Delaunay graph, the maximum number of edges in such a planar graph is 3*N* − 6 [33]. Thus, the total number of switchings leading to increasing the number of edges is at most *M* = (3*N* − 6) − (*N* − 1) = 2*N* − 5, where *N* − 1 corresponds to the number of edges in a minimally connected graph. For the last case as flipping diagonals in Lemma 1, link addition and deletion occur simultaneously so that the number of edges in the interaction topology is invariant. According to the definition of the *r*-limited Delaunay graph, the change of the positional relation among Voronoi cells incurred by the movement of agents causes the switching of the interaction topology. As the velocity of each agent is bounded, it will take a period of time to change the positional relation of nearby Voronoi cells, so that the switching of interaction topologies is not instantaneous but has a dwell time. With the dissipation of the total energy during the dwell time, flipping diagonals will stop in a finite time, which implies that the number of switchings is finite. As a result, the interaction topology of the multi-agent system will keep fixed after a finite number of switchings.

Without loss of generality, we assume that the switching of the interaction topology stops at the time *t*_*k*_. Under the condition of a fixed and connected interaction topology in [*t*_*k*_, ∞), the asymptotic convergence of the velocities of all the agents to the same value is proved by LaSalle’s invariance principle [34]. The level set of *W* with the stacked relative position and velocity vectors is denoted as:

$$\begin{array}{c} \Omega =\{\;\left(\tilde{q}\in D,\tilde{p}\in R^{Nn}\right)|W\left(\tilde{q},\tilde{p}\right)<c\;\}, \end{array}$$ (24)

where

$$\begin{array}{c} D=\{\|{\tilde{q}}_{ij}\|\in R^{N^{2}n}|\|{\tilde{q}}_{ij}\|\in \left(2a,r\right),\;\forall \left(i,j\right)\in E^{\prime }\left(t\right)\}. \end{array}$$ (25)

From the boundedness of the level set, we have $W\leq c\Rightarrow \psi (\|{\tilde{q}}_{ij})\|\leq c$. Likewise, the velocity of each agent satisfies ${\tilde{p}}_{i}^{T}{\tilde{p}}_{i}\leq 2c\Rightarrow \left\|{\tilde{p}}_{i}\right\|\leq \sqrt{2c}$. Due to the connectivity of the interaction topology, there is a path of the length at most *N* − 1 connecting any two agents such as *i* and *j*, so that the distance between them satisfies $\|{\tilde{q}}_{ij}\|<\left(N-1\right)r$. Therefore, the set defined in Eq (24) is a compact set. By LaSalle’s invariance principle, all the trajectories starting in Ω will converge to the largest invariant set inside the region

$$\begin{array}{c} S=\{\;\tilde{q}\in D,\tilde{p}\in R^{Nn}|\dot{W}=0\;\}, \end{array}$$ (26)

which implies that $-{\tilde{p}}^{T}\left(L_{G_{LD}\left(r\right)}⊗I_{n}\right)\tilde{p}=0$. Since $G_{LD}\left(r\right)$ is connected, the positive semi-definite Laplacian matrix $L_{G_{LD}\left(r\right)}$ has an eigenvalue 0 corresponding to a positive eigenvector **1**^*T*^ = {1, …, 1}^*T*^. Solving the algebraic equation related to the invariance set, we obtain that $\tilde{p}=c1$, where *c* is a constant. The result implies that ${\tilde{p}}_{1}=\cdots ={\tilde{p}}_{N}$, i.e., the velocity vectors of all the agents converge to the same value as ‖*p*_*i*_ − *p*_*j*_‖ → 0 for all *i* ≠ *j* as *t* → ∞.

According to velocity consensus, statement 3 is proved by the fact that $\dot{\tilde{p}}=0$, which means

$$\begin{array}{c} {}[\begin{array}{c} -\sum_{j\in N_{1}^{LD}\left(r\right)}\nabla_{q_{1}}\psi (\|{\tilde{q}}_{ij}\left\|\right)\\ \vdots \\ -\sum_{j\in N_{N}^{LD}\left(r\right)}\nabla_{q_{N}}\psi (\|{\tilde{q}}_{ij}\left\|\right) \end{array}]=0. \end{array}$$ (27)

Its solution corresponds to a local minimum of the collective potential function of each agent. Under this condition, relative distances between one agent and its neighbors become constant. Due to the planarity of the *r*-limited Delaunay graph, there are no intersected edges in this graph. Therefore, the final configuration of the multi-agent system converges asymptotically to a quasi-lattice formation, in which the distance between any two adjacent agents tends to be fixed and approaches the desired distance at which the potential function *ψ*(⋅) obtains its minimum, i.e., the solution of the equation $\nabla_{q_{i}}\psi (\|{\tilde{q}}_{ij}\left\|\right)=0$.

Finally, because of the boundedness of *W*(*t*) for all *t* > 0 and the fact that $\lim_{\|{\tilde{q}}_{ij}\|\to 2a}\psi (\|{\tilde{q}}_{ij}\left\|\right)=\infty$, collisions among agents are avoided. This completes the proof of statement 4.

*Remark* 3. Unlike the flocking algorithm proposed in [19], where a quasi-lattice formation is obtained by imposing the constraint on the ratio of agent’s sensing range to the desired distance between two adjacent agents, the final configuration of the multi-agent system steered by our flocking algorithm spontaneously becomes a quasi-lattice formation due to the planarity of the *r*-limited Delaunay graph as the interaction topology.

## 4 Simulations

In this section, we compare the proposed flocking algorithm with that proposed in [14], where the *r*-disk graph is applied to represent the interaction topology of the multi-agent system as well as the flocking algorithm based on the Delaunay graph as its interaction topology. The correctness and effectiveness of our flocking algorithm are verified through extensive numerical simulations in three scenarios, where the initial configurations of a multi-agent system are different. For a multi-agent system consisting of 50 agents having the same sensing range *r* = 1 and collision shell *a* = 0.01, the initial configurations includes a random, linear and circular formation in the plane, respectively. In the meanwhile, the initial velocity vectors of agents are randomly chosen in the unit square. The value of the hysteresis in link addition *ε* is set to 0.0001, smaller than the upper bound ε* ≈ 0.0002, which is derived from Eq (17).

**Fig 7:**
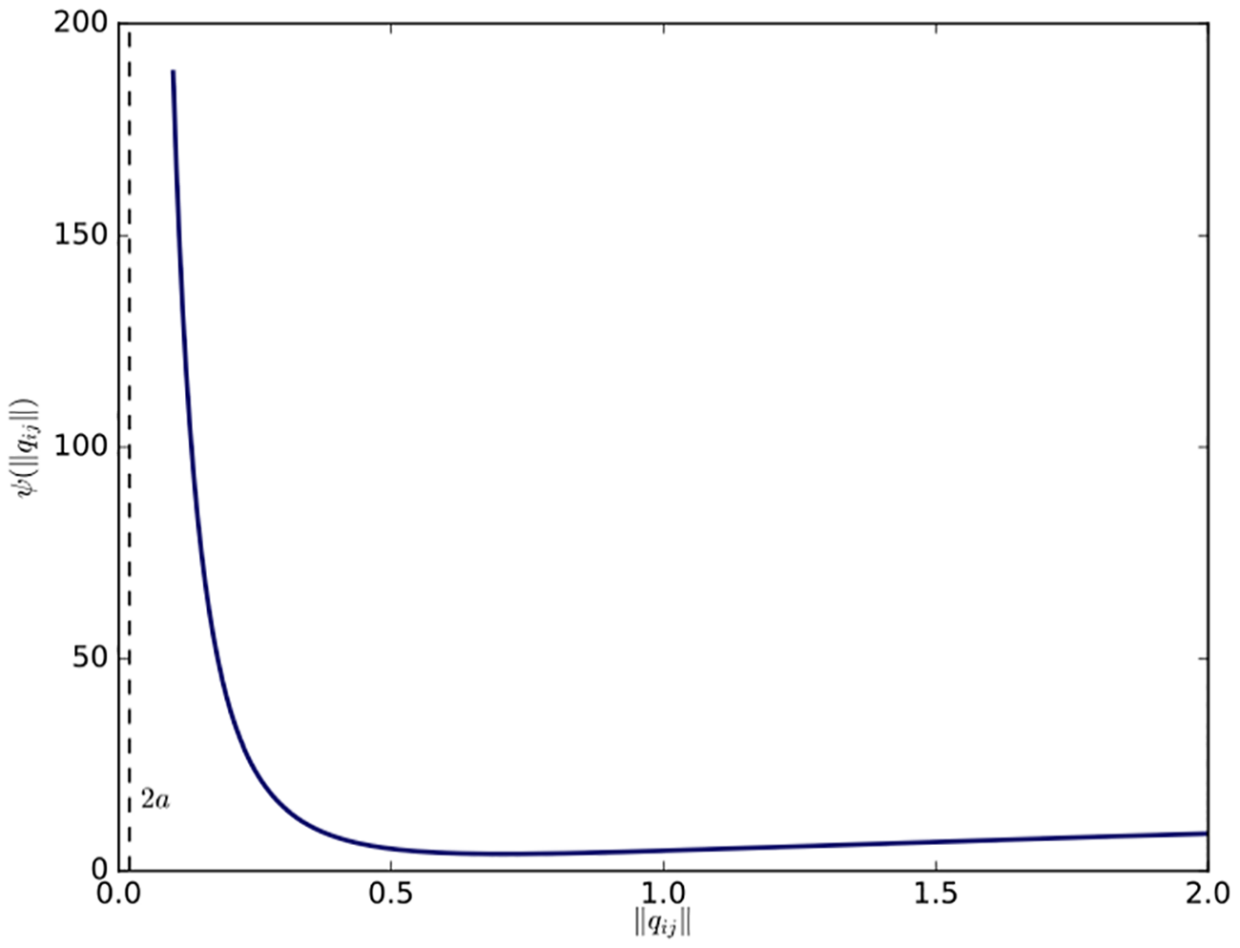
Smooth potential function. The smooth potential function without the constraint on the sensing range.

**Fig 8:**
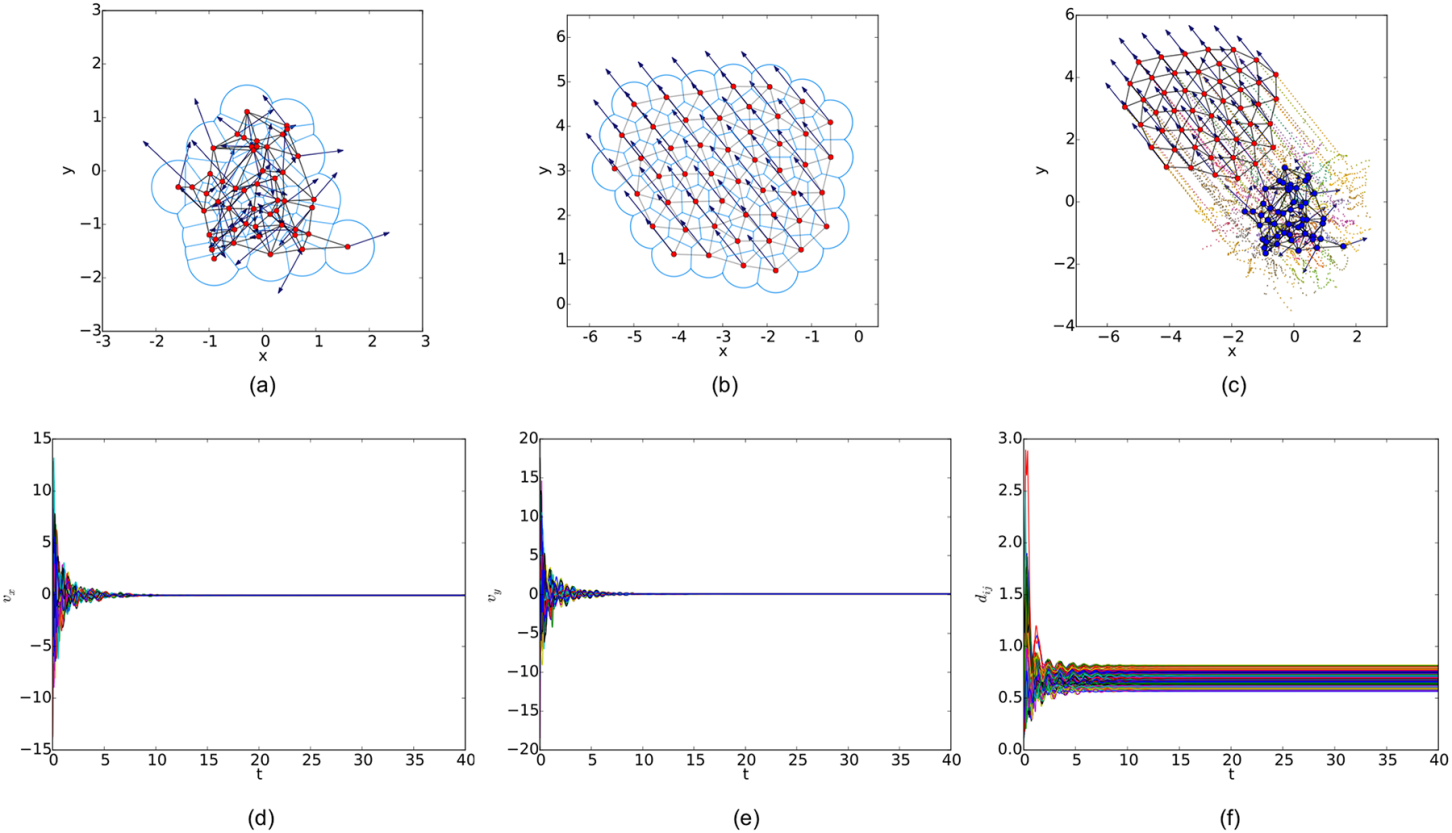
Simulation results of the proposed flocking algorithm with a random initial configuration. (a) initial configuration. (b) final configuration. (c) evolution of the multi-agent system. (d) consensus of velocity in *x* component. (e) consensus of velocity in *y* component. (f) variations of relative distances between neighbors.

**Fig 9:**
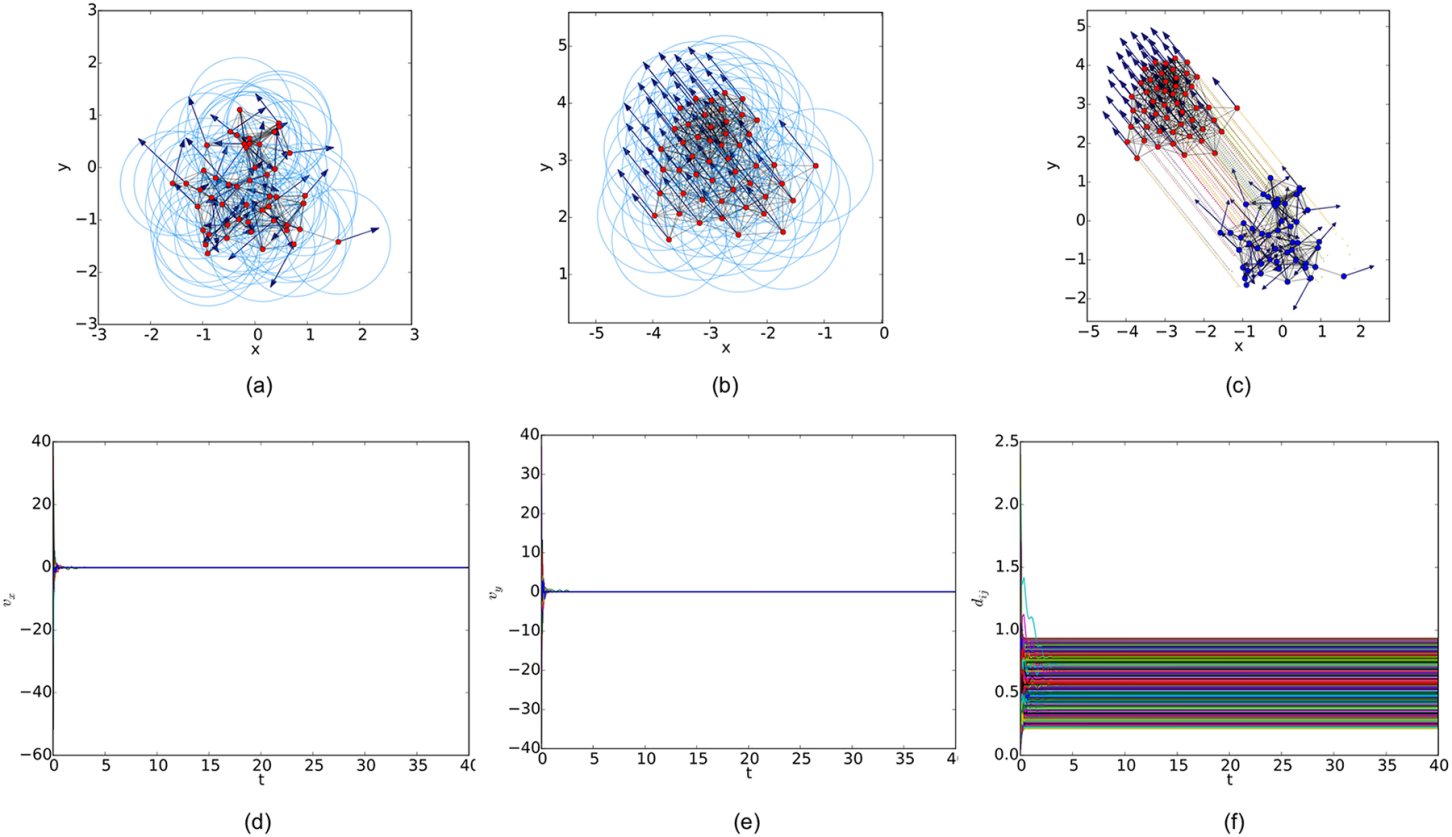
Simulation results of the flocking algorithm proposed in [14] with a random initial configuration. (a) initial configuration. (b) final configuration. (c) evolution of the multi-agent system. (d) consensus of velocity in *x* component. (e) consensus of velocity in *y* component. (f) variations of relative distances between neighbors.

**Fig 10:**
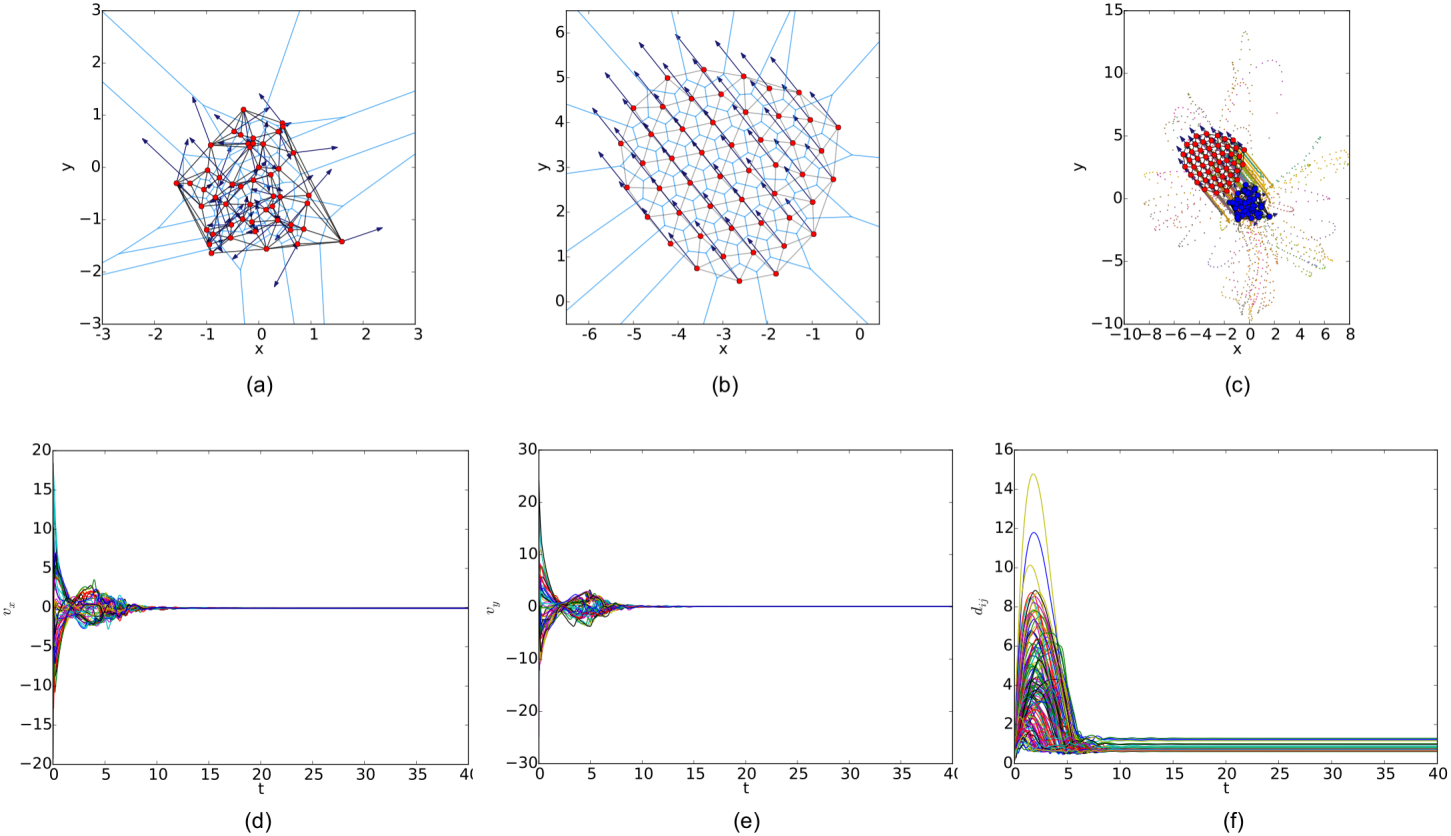
Simulation results of the flocking algorithm based on the Delaunay graph with a random initial configuration. (a) initial configuration. (b) final configuration. (c) evolution of the multi-agent system. (d) consensus of velocity in *x* component. (e) consensus of velocity in *y* component. (f) variations of relative distances between neighbors.

For a random initial configuration, the initialization procedure used to generate a collision-free and cohesive formation is given as follows: first, establish a Cartesian coordinates system and deploy an agent at the origin, then place the next agent in the annulus between 2*a* and *r* − *ε* of any existing agent. Finally, this step continues until the specified number of agents is reached. Therefore, a connected *r*-disk graph is generated and the corresponding *r*-limited Delaunay graph can be computed from it as a feasible interaction topology. In the meanwhile, any configuration of the multi-agent system will lead to a connected Delaunay graph since the constraint on the sensing range is ignored in this graph. Therefore, a different potential function like the one applied in [11] (shown in Fig 7) is used in our simulations. The simulation results of the three flocking algorithms under the random initial condition are shown in Figs 8, 9 and 10, respectively. The initial configurations represented as the three different proximity graphs are shown in Figs 8(a) and 9(a) and 10(a), respectively, where the red solid dots indicate agents, deep blue arrows attached to agents indicate their velocity vectors (arrows are scaled with a proper factor for a clear illustration), regions enclosed by light blue arcs and/or line segments are the distinct dominant regions of each agent and grey line segments between any two agents are edges in the interaction topology. The final states of the multi-agent system shown in Figs 8(b), 9(b) and 10(b) imply that all the three flocking algorithms can drive the multi-agent system to flocking motion. In Figs 8(c), 9(c) and 10(c), the evolutions of the multi-agent system are produced by interpolating the moving trajectories of agents between their initial and final positions with colored dots. The velocity curves of all the agents are shown in Figs 8(d), 9(d) and 10(d) for *x* component and Figs 8(e), 9(e) and 10(e) for *y* component, respectively, where they finally converge to the same value. According to the edge set in the final interaction topology, the variations of relative distances between two adjacent agents are shown in Figs 8(f), 9(f) and 10(f) respectively, where they tend to be constant as the system evolves.

**Fig 11:**
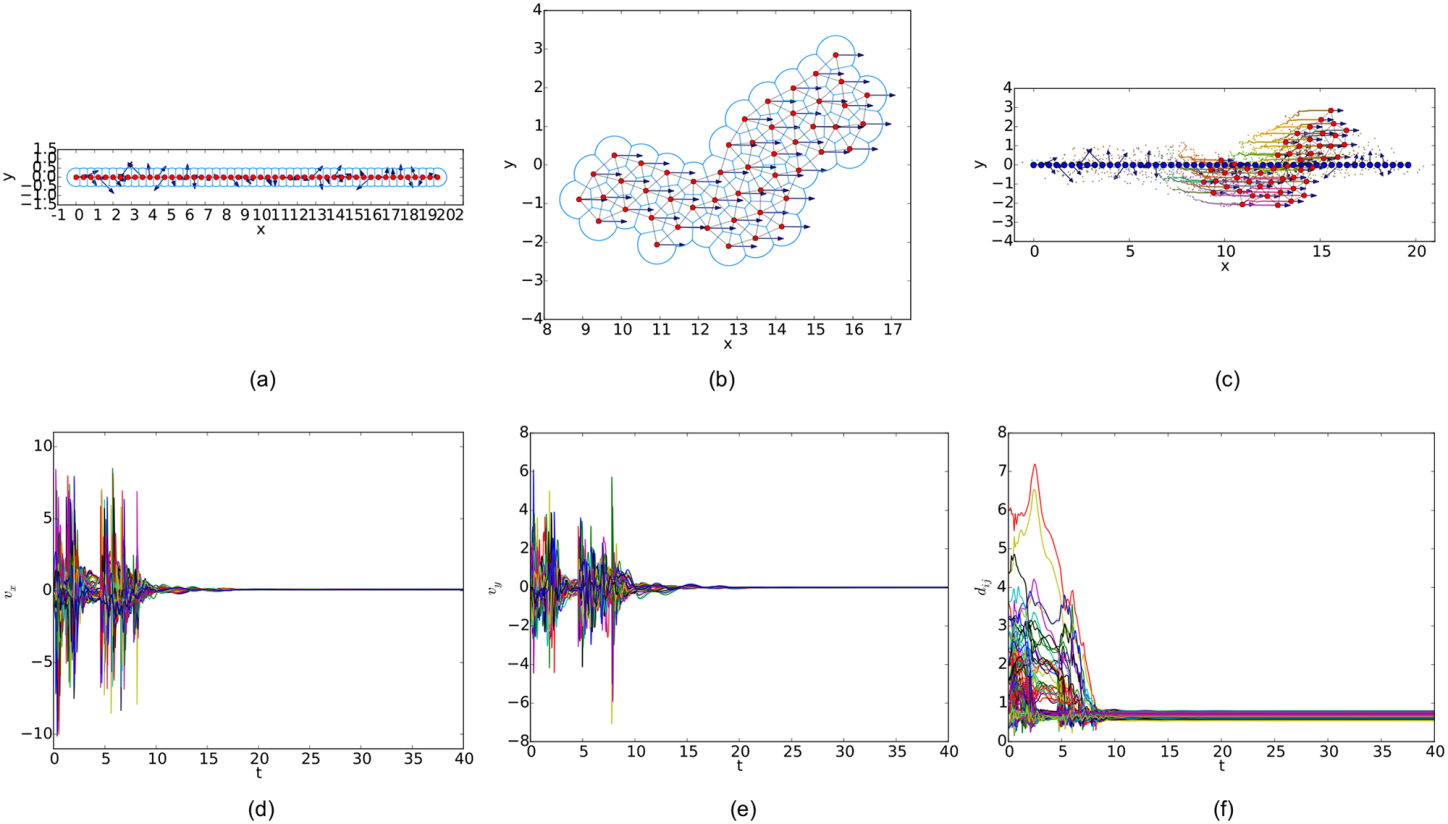
Simulation results of the proposed flocking algorithm with a linear initial configuration. (a) initial configuration. (b) final configuration. (c) evolution of the multi-agent system. (d) consensus of velocity in *x* component. (e) consensus of velocity in *y* component. (f) variations of relative distances between neighbors.

**Fig 12:**
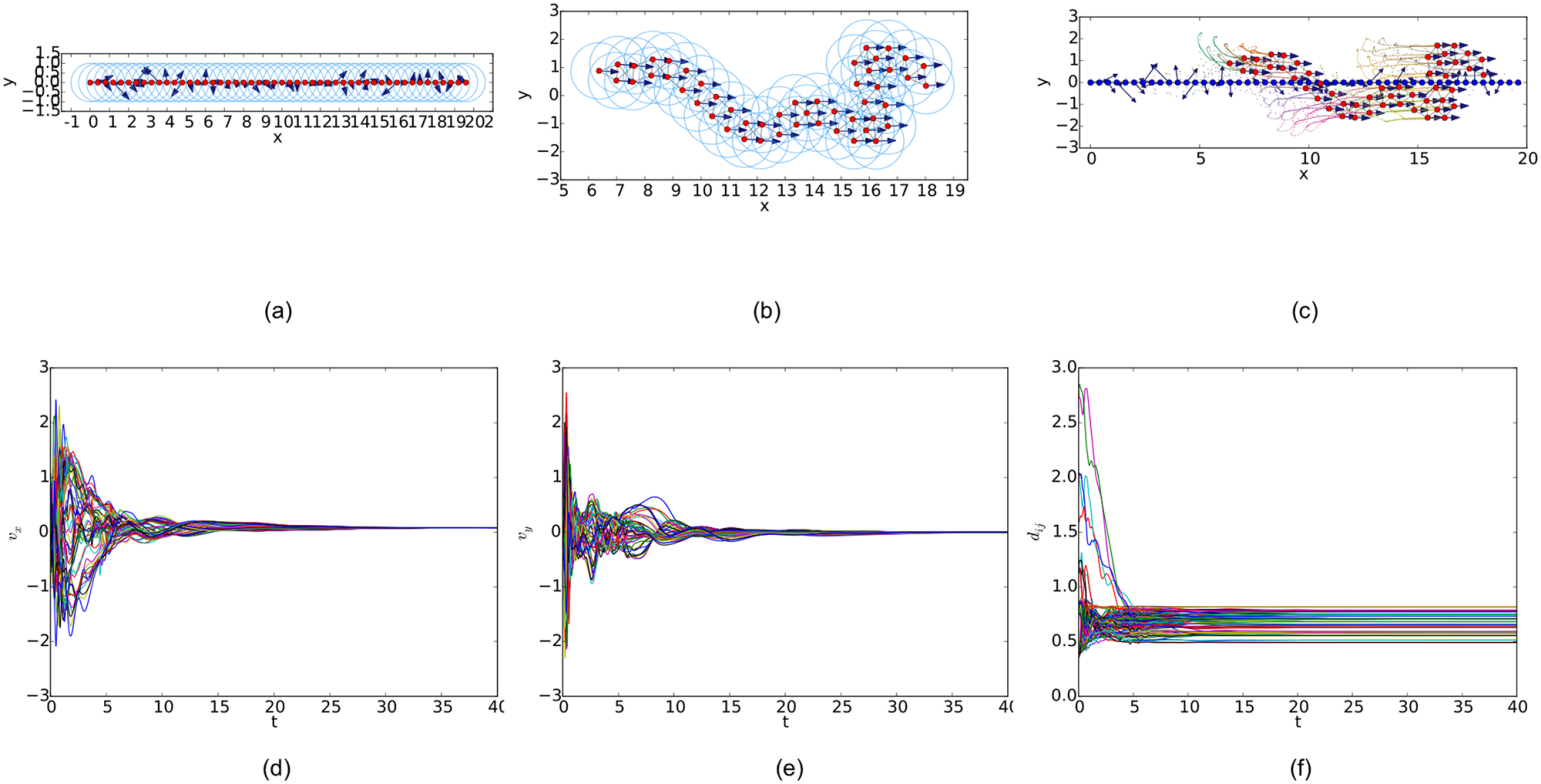
Simulation results of the flocking algorithm proposed in [14] with a linear initial configuration. (a) initial configuration. (b) final configuration. (c) evolution of the multi-agent system. (d) consensus of velocity in *x* component. (e) consensus of velocity in *y* component. (f) variations of relative distances between neighbors.

**Fig 13:**
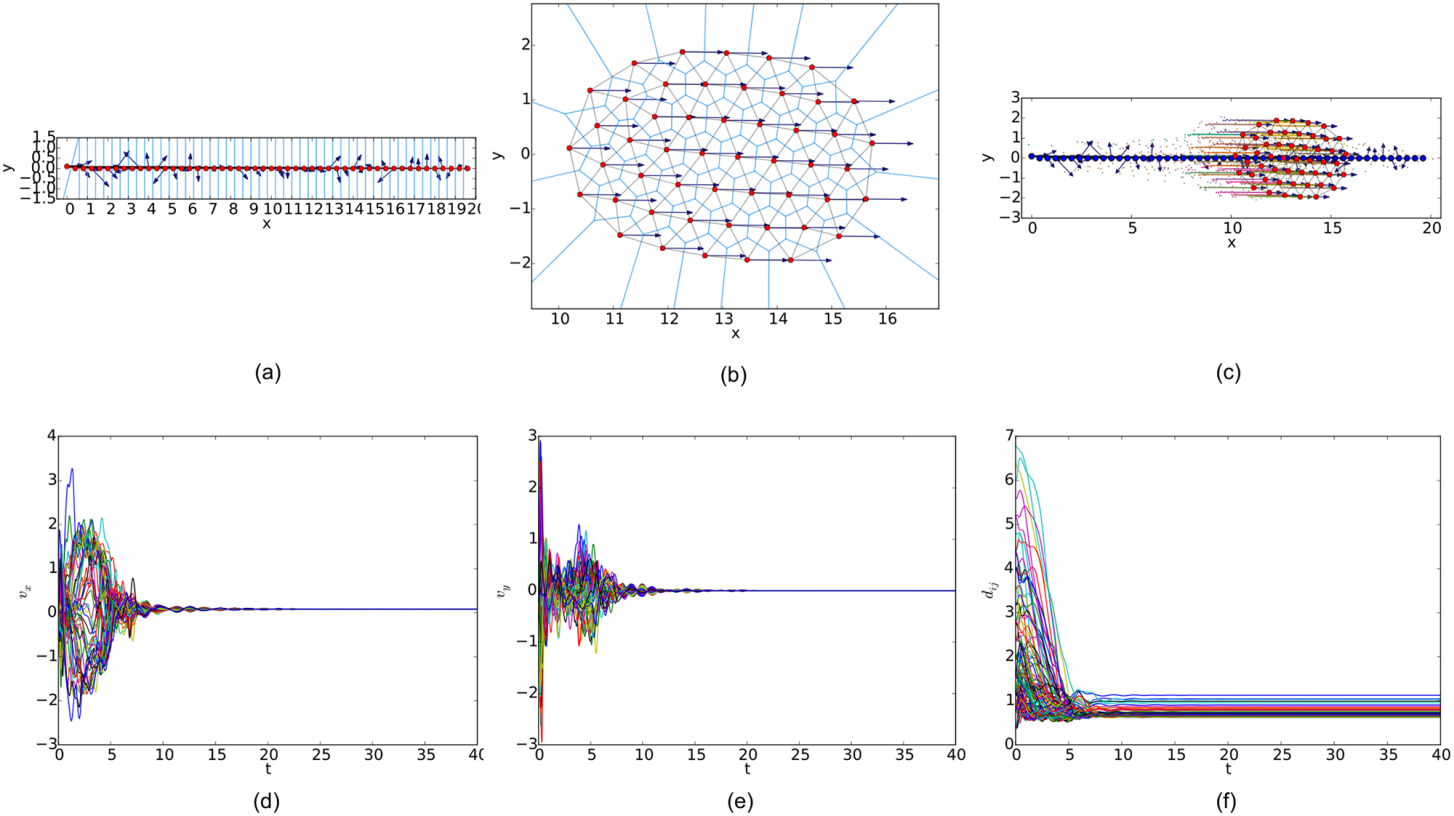
Simulation results of the flocking algorithm based on the Delaunay graph with a linear initial configuration. (a) initial configuration. (b) final configuration. (c) evolution of the multi-agent system. (d) consensus of velocity in *x* component. (e) consensus of velocity in *y* component. (f) variations of relative distances between neighbors.

**Fig 14:**
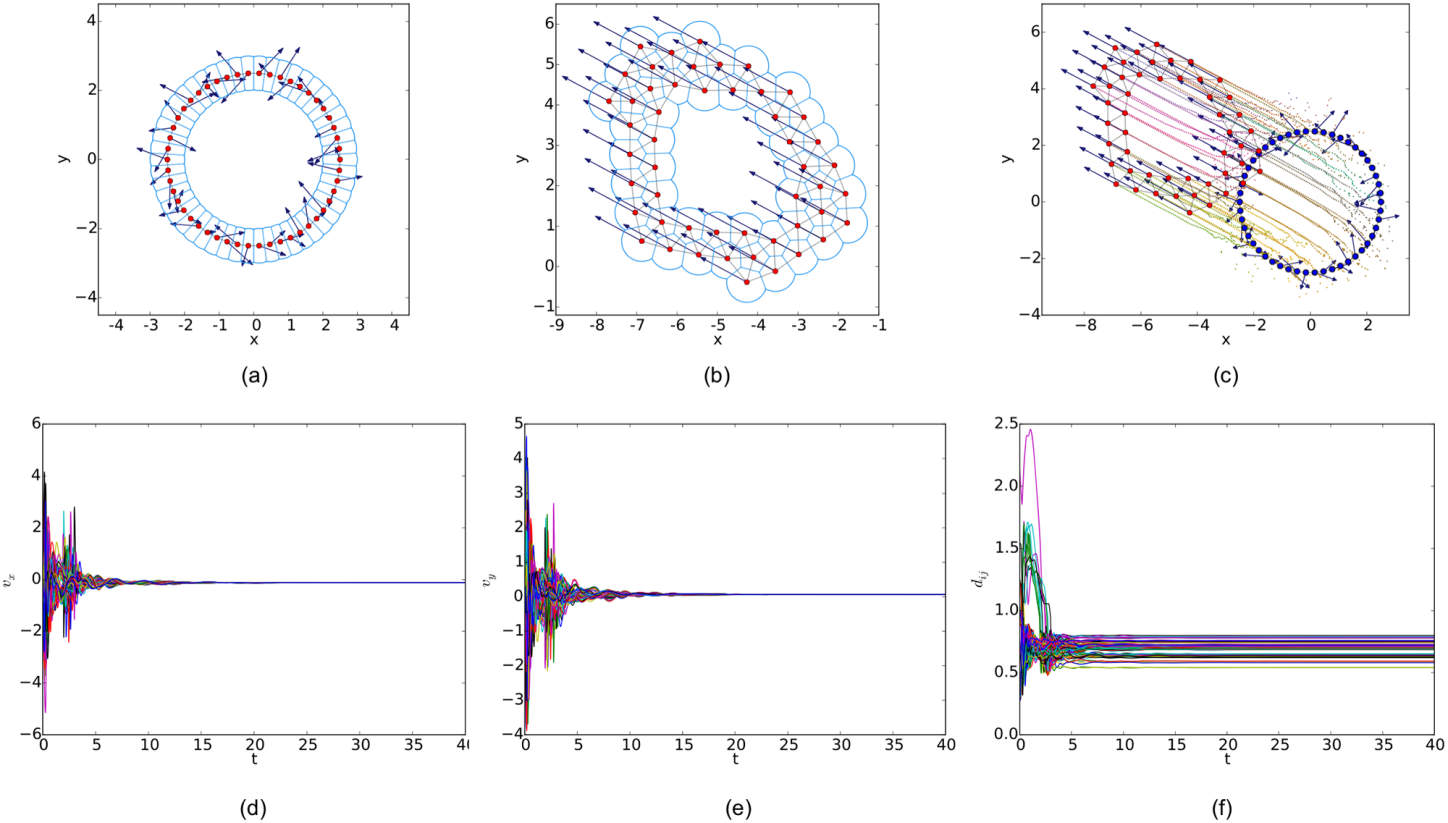
Simulation results of the proposed flocking algorithm with a circular initial configuration. (a) initial configuration. (b) final configuration. (c) evolution of the multi-agent system. (d) consensus of velocity in *x* component. (e) consensus of velocity in *y* component. (f) variations of relative distances between neighbors.

**Fig 15:**
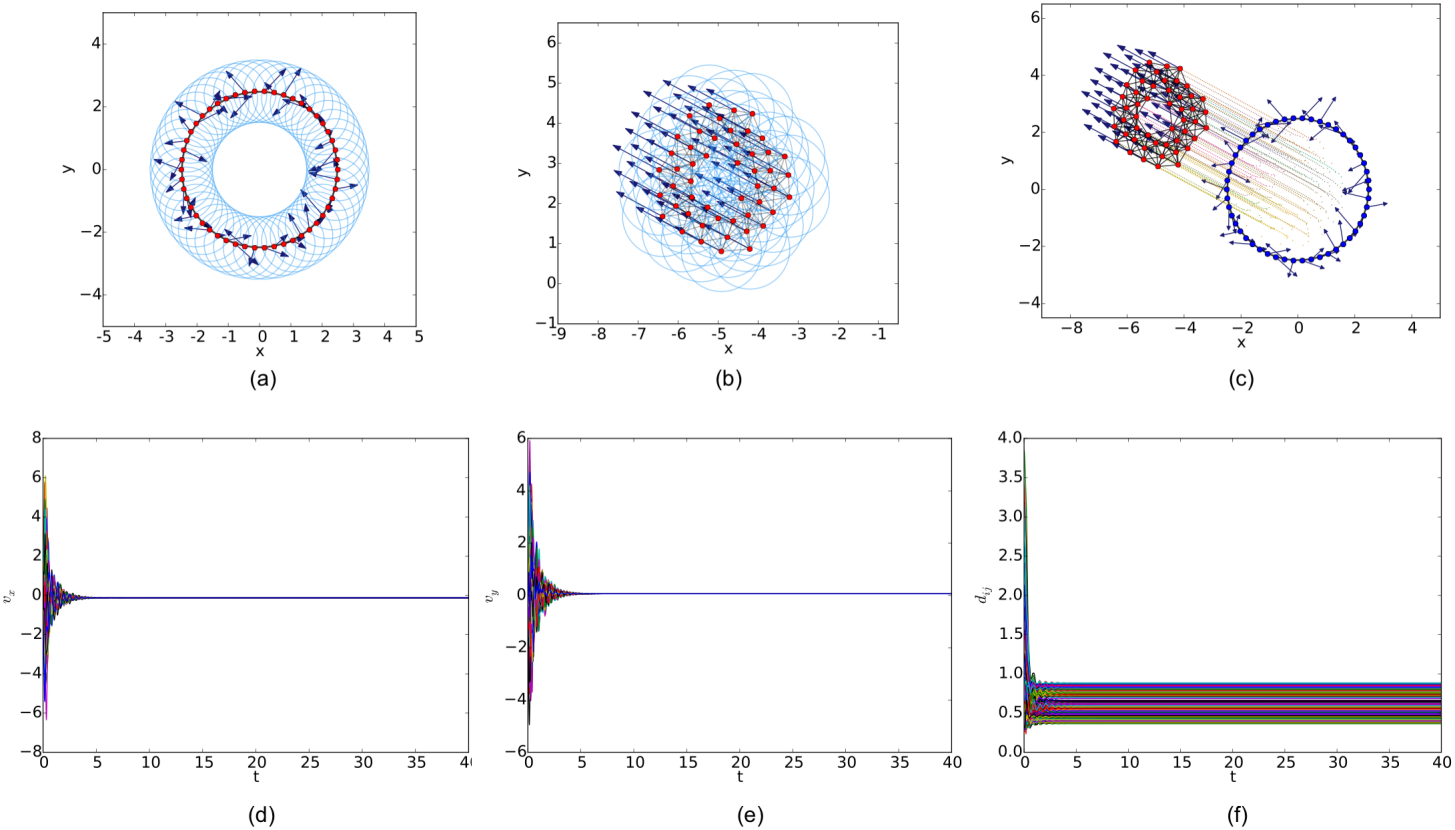
Simulation results of the flocking algorithm proposed in [14] with a circular initial configuration. (a) initial configuration. (b) final configuration. (c) evolution of the multi-agent system. (d) consensus of velocity in *x* component. (e) consensus of velocity in *y* component. (f) variations of relative distances between neighbors.

**Fig 16:**
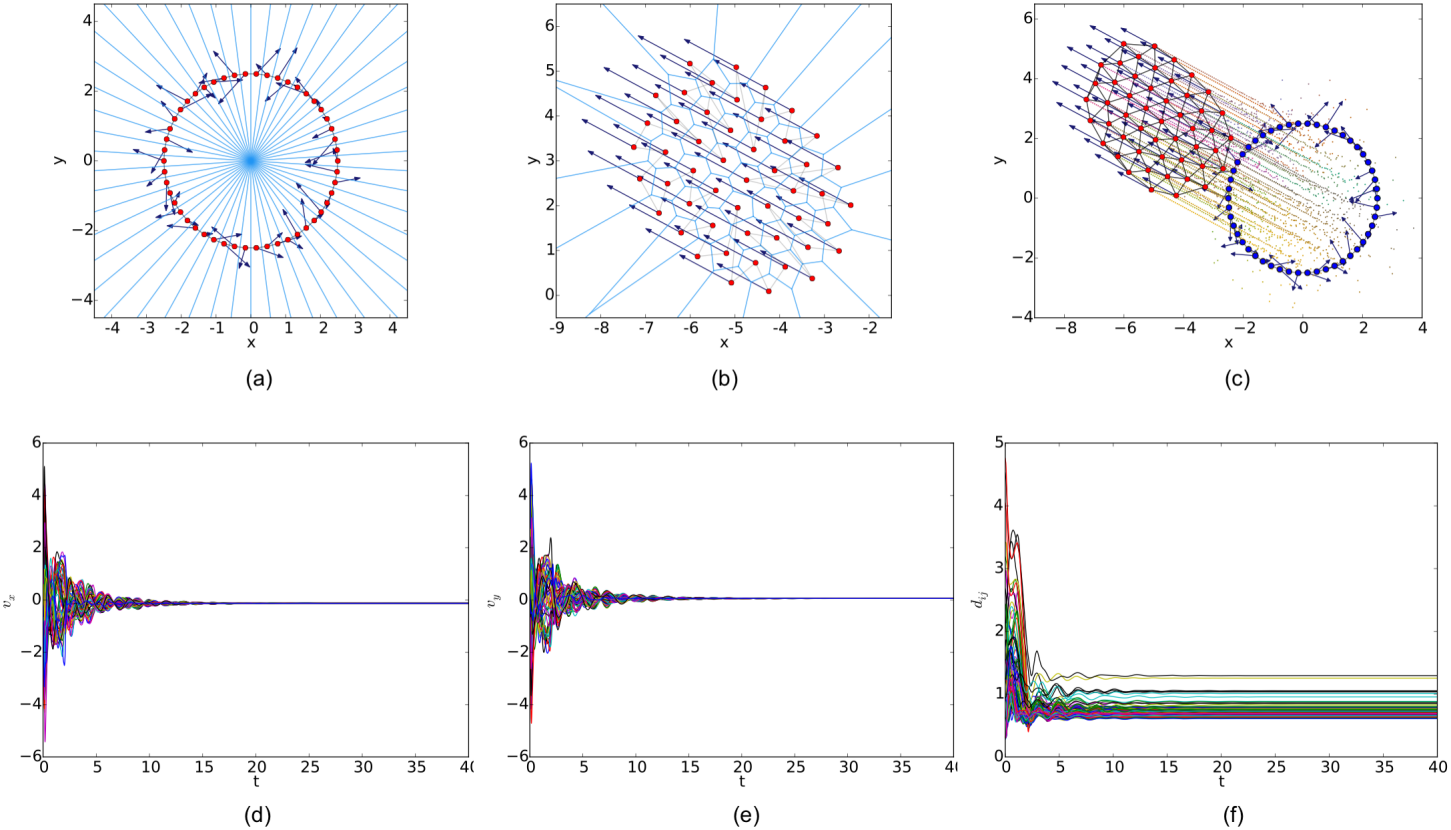
Simulation results of the flocking algorithm based on the Delaunay graph with a circular initial configuration. (a) initial configuration. (b) final configuration. (c) evolution of the multi-agent system. (d) consensus of velocity in *x* component. (e) consensus of velocity in *y* component. (f) variations of relative distances between neighbors.

Similarly, simulation results from a linear initial configuration are illustrated in Figs 11, 12 and 13, where all the agents are deployed on a horizontal line with distances between adjacent agents equal to 0.4. At last, simulation results from a circular initial configuration are shown in Figs 14, 15 and 16, where agents are uniformly distributed on the circumference of a circle with the radius equal to 2.5.

By comparing the three flocking algorithms with three different initial configurations, all the aforementioned advantages of our flocking algorithm are demonstrated. Firstly, the number of edges in the *r*-limited Delaunay graph is much less than that in the *r*-disk graph and almost equal to that in the Delaunay graph. For example, the number of edges |E(t)| as the interaction complexity in the final interaction topology shown in Figs 8(b), 9(b) and 10(b) are 128, 431 and 132, respectively. It is obvious that the cost of the information exchange among agents in the *r*-limited Delaunay graph is almost the same as that in the Delaunay graph and greatly lower than that in the *r*-disk graph. Secondly, during the evolution process of the multi-agent system steered by our flocking algorithm, link deletion is allowed without destroying the connectivity of the *r*-limited Delaunay graph and Delaunay graph, for instance, the edge between agent 2 and 16 in Fig 8(c) and 8 and 17 in Fig 10(c). On the contrary, link deletion cannot occur in the evolution process of the multi-agent system steered by the flocking algorithm proposed in [14]. It implies that our flocking algorithm retains the fine property derived from the Delaunay graph and is more flexible than that based on the *r*-disk graph since some unnecessary links which may hinder the realization of flocking motion can be dynamically disconnected. Finally, relative distances between two adjacent agents in the final configuration generated by our flocking algorithm are much closer to the desired distance (see Fig 8(f)). In contrast, relative distances generated by the compared algorithm in [14] have a large deviation from the desired value and distribute more dispersedly (see Fig 9(f)). It is shown that our flocking algorithm can lead the multi-agent system to a more regular quasi-lattice formation as in Fig 10(f) obtained from the Delaunay graph.

## 5 Conclusion

In this paper, we propose a novel connectivity-preserving flocking algorithm, where the *r*-limited Delaunay graph rather than the commonly used *r*-disk or Delaunay graph is used to represent the interaction topology of a multi-agent system. As a result, our flocking algorithm has a low computational complexity. The mechanism of link deletion brings the flexibility into flocking motion of the multi-agent system. The regular quasi-lattice formation is generated spontaneously without extra constraints. Moreover, our flocking algorithm can be implemented in a distributed fashion.

Since the infinite potential function applied in our algorithm is impractical for real systems, we will use the finite potential function to improve our flocking algorithm in the future work. Furthermore, other types of proximity graphs with different properties will be used to represent the interaction topology of a multi-agent system so as to generate various kinds of formations.
